# Red blood cells in biology and translational medicine: natural vehicle inspires new biomedical applications

**DOI:** 10.7150/thno.87425

**Published:** 2024-01-01

**Authors:** Xueyun Zhang, Yindan Lin, Jinxia Xin, Ying Zhang, Kaiwen Yang, Yan Luo, Ben Wang

**Affiliations:** 1 Cancer Institute (Key Laboratory of Cancer Prevention and Intervention, China National Ministry of Education), The Second Affiliated Hospital, Zhejiang University School of Medicine, Hangzhou, China, 310009.; 2 Department of Biochemistry, Zhejiang University School of Medicine, Hangzhou, China, 310058.; 3 Department of Biochemistry & Cancer Medicine, International Institutes of Medicine, the Fourth Affiliated Hospital of Zhejiang University School of Medicine, Yiwu, Zhejiang, China.; 4 Institute of Translational Medicine, Zhejiang University, Hangzhou, China, 310029.; 5 Cancer Center, Zhejiang University, Hangzhou, China, 310029.; 6 Hon Hai Precision Industry Co., Ltd.

**Keywords:** cell surface engineering, cell therapy, drug delivery, red blood cells, universal blood

## Abstract

Red blood cells (RBCs) are the most abundant cell type in the blood, and play a critical role in oxygen transport. With the development of nanobiotechnology and synthetic biology, scientists have found multiple ways to take advantage of the characteristics of RBCs, such as their long circulation time, to construct universal RBCs, develop drug delivery systems, and transform cell therapies for cancer and other diseases. This article reviews the component and aging mystery of RBCs, the methods for the applied universal RBCs, and the application prospects of RBCs, such as the engineering modification of RBCs used in cytopharmaceuticals for drug delivery and immunotherapy. Finally, we summarize some perspectives on the biological features of RBCs and provide further insights into translational medicine.

## Introduction

Mature human red blood cells (RBCs) are well-known for their biconcave disk shape. They contain no organelles or complete energy or genetic system, unlike normal nucleated mammalian cells. However, they have a long lifespan of approximately 120 days, traveling through arteries, veins, and capillary vessels in the circulatory system to deliver oxygen to tissues for gas exchange.

The unique properties of RBCs are essential for their function in the body. The biconcave shape of RBCs is a result of their lack of a nucleus and their high concentration of hemoglobin. The biconcave shape allows RBCs to squeeze through narrow capillaries by two mechanisms: flexibility and thinness. The flexibility of the RBC membrane allows it to deform and change shape as it passes through narrow capillaries. The thinness of the RBC membrane also helps it to squeeze through narrow capillaries. This is essential for the delivery of oxygen and nutrients to all parts of the body. What's more, the biconcave shape of RBCs also helps to increase their surface area to volume ratio. The surface area to volume ratio of a sphere is equal to 4πr²/3. The surface area to volume ratio of a biconcave disk is equal to 8πr²/3, which is greater than that of a sphere. This is important for gas exchange, as it allows the RBCs to have more surface area for oxygen and carbon dioxide to diffuse across. This is why RBCs have a biconcave shape. The increased surface area to volume ratio of RBCs allows them to carry more oxygen and carbon dioxide per unit volume. Moreover, the biconcave shape also allows RBCs to stack together in a way that minimizes their resistance to flow. This is important for the efficient transport of oxygen and nutrients throughout the body.

The cell membrane, the only structural component of erythrocytes, accounts for all of their diverse antigenic, transport, and mechanical characteristics 1. The cell membrane is composed of a lipid bilayer, with proteins embedded in both layers. The proteins on the cell membrane provide RBCs with their antigenic properties, while the proteins in the lipid bilayers which are called hemoglobin, help to transport oxygen and carbon dioxide across the membrane. The biogenetic and mature mechanisms of erythrocytes have been investigated quite thoroughly 2,which involve the development of RBCs from stem cells in the bone marrow, and the mature mechanisms of erythrocytes involve the functions of RBCs in the bloodstream. The spleen and liver play key roles in erythrocyte metabolism. The spleen filters out old and damaged RBCs, while the liver recycles the iron from old RBCs. The spleen also produces antibodies that help to protect against infection. Bessis and Delpech *et al.* (1981) reviewed the priorities and credits for the discovery of RBCs 3.

Universal red blood cells (U-RBCs) are a type of RBCs that is compatible with blood from all donors, regardless of their blood type. This makes them a potentially valuable resource for blood transfusions 4, and could help to solve the problem of blood shortages and improve the safety of blood transfusions. U-RBCs are being developed based on the fundamental structure and function of RBCs. There are several different approaches to developing U-RBCs, including removing the antigens that are responsible for incompatibility; masking the antigens on the surface of RBCs so that they are not recognized by the recipient's immune system; developing artificial RBCs that do not have any antigens. There are many groups that did research on U-RBCs and made plenty of promising progress 5. This review summarizes the work progress on developing U-RBCs, including the modification of antigen enzymes, masking of blood group antigens, *ex vivo* synthesis, and artificial RBCs.

It's a promising platform of RBCs for drug delivery due to their unique properties, such as their long circulation time in the bloodstream, their ability to target specific tissues, and their biodegradability. RBCs can be loaded with drugs in a number of ways, such as encapsulating the drug in a polymer coating; linking the drug to the RBC membrane; transfecting the RBC with genes that encode for the drug. Once the drug is loaded into the RBC, it can be released slowly over time, or it can be released in a targeted manner.

The use of RBCs for drug delivery has several advantages. First, RBCs' abundant and long circulation time in the bloodstream allows for a slow and sustained release of the drug. Second, easily accessible and with the ability to target specific tissues, RBCs can be used to deliver drugs to a variety of tissues, including the brain, liver, and tumors. They can also be used to deliver drugs to specific cells, such as cancer cells or immune cells. Third, RBCs are biodegradable, which means that they are broken down by the body after they have released their payload.

However, RBCs still have a limited lifespan after which they are removed from circulation by the spleen and liver. The process of RBC aging and clearance is essential for maintaining the health of the blood and preventing the accumulation of damaged RBCs, which can lead to a few health problems, including anemia, jaundice, and kidney disease. Free radicals, glycation, and apoptosis are common factors that contribute to the aging of normal cells and RBCs, while the blood is a very complex mixture of many different components, plasma, RBCs, WBCs, and platelets, electrolytes, hormones, and waste products, which made the aging and clearance process of RBCs more complex, what's more, natural antibodies, immunoglobulin G, opsonin, and the complement system, have all been proven to contribute to this process 6. Some membrane disorders are also involved in the RBC aging and clearance process, such as band 3 clustering, phosphatidylserine (PS) exposure, the denaturation of CD47, and the cracking-off of sialic acid. In 2015, some new aspects have been taken into consideration, such as cell deformability and shape 7. The aging and clearance process is not only playing important roles in naturally mature RBCs but also critical for drug-loaded RBCs as it can affect the RBC property, shape, and circulation life *in vivo*.

In this review, we discuss the mechanisms of nature-made long-circulating vehicles (RBCs) from physiochemical and biological perspectives. We also provide insights and inspiration for the development of universal red cells, man-made drug delivery systems, and cancer immunotherapy for translational applications in clinical settings.

## The molecular structure of RBC membranes

**Figure 1:**
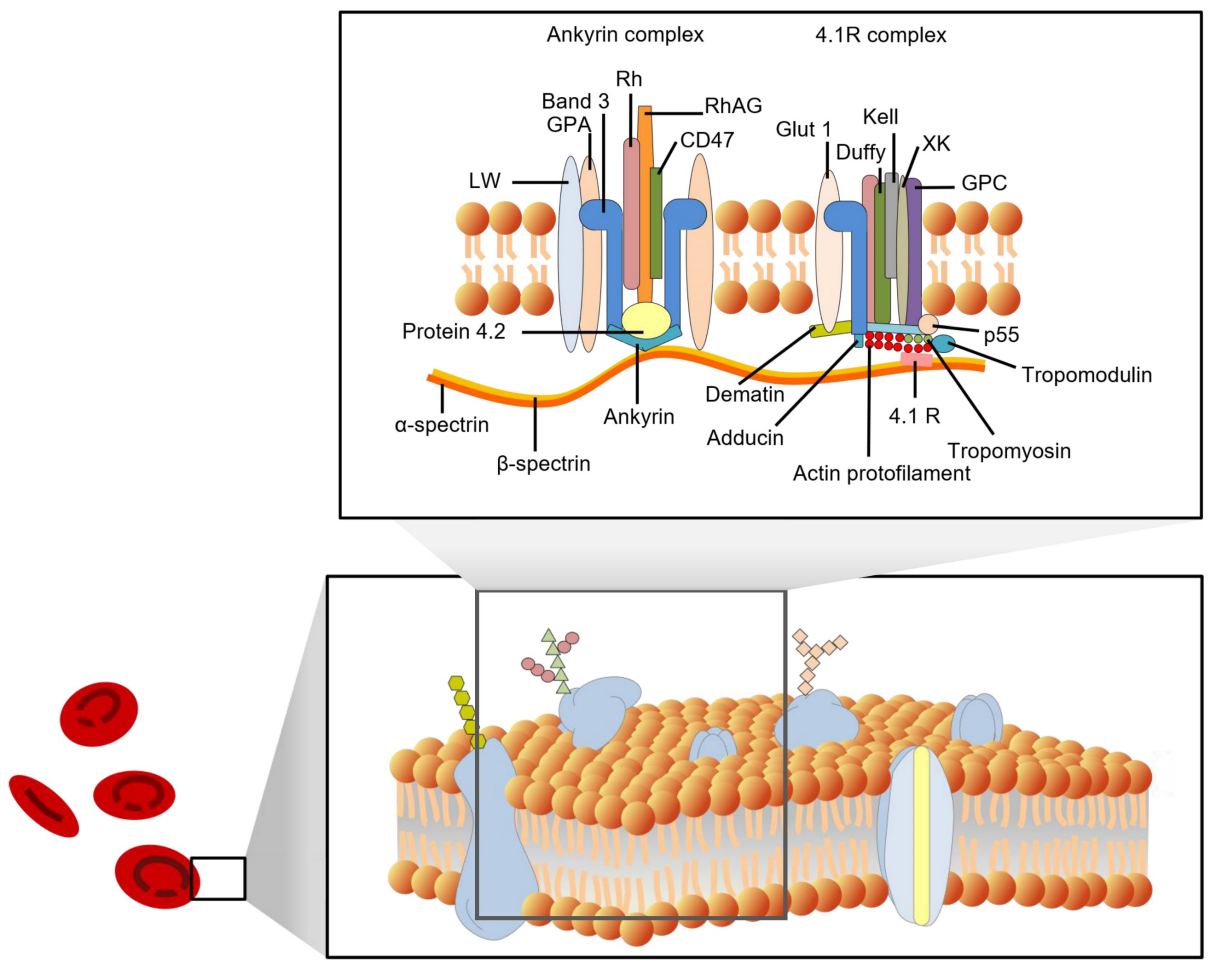
A schematic representation of a red cell membrane. The plasma membrane is a composite structure composed of amphiphilic lipid molecules. A two-dimensional elastic network of skeletal proteins is embedded in the lipid bilayer through tethering sites (transmembrane proteins).

In recent decades, many fundamental discoveries have been made about the structure of cell membranes. In 1972, Singer and Nicolson proposed the fluid mosaic model of the cell membrane 8. Four years later, Marchesi and Furthmayr published a comprehensive review of the membrane of RBCs 9. In addition to the lipid bilayer, RBC membranes also contain integrin and peripheral membrane proteins, as well as carbohydrates that are embedded in the inner leaflet. With the development of new techniques, such as freeze-fracture microscopy, scanning probe microscopy (SPM), and X-ray diffraction, the structure of RBC membranes has become increasingly clear. Figure 1 shows some details of the red cell membrane.

### Lipids

Approximately 40% of the weight of the RBC membrane is composed of lipids. The main types of lipids are phospholipids (24%), unesterified cholesterol (12%), and glycerides and free fatty acids (4%). Cholesterol is an essential structural component of the RBC membrane, controlling its surface area, structural integrity, and fluidity. This allows RBCs to change shape and move around.

### Membrane proteins

Mark S. Bretscher et al. (1973) summarized the general principles of membrane structure and three of the major protein components of the erythrocyte membrane in their review 10. The three components were separated based on molecular weight using sodium dodecyl sulfate-polyacrylamide gel electrophoresis (SDS-PAGE). Tektin A, which was separated from blood ghosts and named by Clarke et al. (1971), has two chains (alpha and beta) with molecular weights of 220 kDa and 240 kDa, respectively 11. These chains are known as spectrin or band 1 (alpha chain) and band 2 (beta chain). Component A (100 kDa), also called band 3, is exposed on the surface of erythrocytes and functions in anion exchange. The major blood type epitopes are glycoproteins A, B, and C, which have molecular weights of approximately 30 kDa. These glycoproteins are located on the surface of RBCs and are recognized by the immune system to determine blood type. Blood types such as A, B, and O are determined by the presence or absence of these glycoproteins. There are many other proteins, such as band 4.1, with a molecular weight of approximately 83 kDa, and actin is approximately 43 kDa, and it's a member of the membrane skeleton. Some proteins cannot be recognized due to their low content.

According to their position on the cell membrane, these proteins can be divided into integrated and peripheral membrane proteins. Integral membrane proteins are proteins that are embedded in the lipid bilayer of cell membranes. They are held in place by hydrophobic interactions with the lipid molecules, and they play a variety of important roles in cell function, including transport of molecules across the membrane, signaling, and energy transduction 12. They include band 3 protein (anion exchanger 1, AE1), glycophorins (glycophorins A, B, and C), Na^+^/K^+^ ATPase, band 4.5 protein (glucose transport protein), and surface receptors; Peripheral membrane proteins are not embedded in the membrane, but are associated with it through non-covalent interactions. They include spectrin (bands 1 and 2), ankyrin (bands 2.1, 2.2, and 2.3), band 4.1, and actin (band 5). The peripheral proteins are components of the cell cytoskeleton. Spectrin is a major component of the cytoskeleton, and it forms a network of interconnected rods that help to maintain the shape of the cell membrane. Ankyrin binds to spectrin and anchors it to the membrane. Band 4.1 also binds to spectrin and helps to stabilize the cytoskeleton. Actin is a small protein that also plays a role in the cytoskeleton. The interactions between the peripheral proteins are mediated by band 4.2. Band 4.2 binds to both spectrin and ankyrin, and helps to link them together. This helps to form a strong and stable network that supports the cell membrane. Figure 1 shows a schematic diagram of the interactions between the peripheral membrane proteins.

The membrane proteins in RBCs have a variety of functions, such as transporting oxygen and carbon dioxide, maintaining the shape of RBCs, protecting the RBCs from damage, and helping to identify RBCs. Hemoglobins help to transport oxygen and carbon dioxide across the cell membrane. Other membrane proteins, such as actin, band 4.1, spectrin, ankyrin, and band 3, help maintain RBCs' biconcave shape. The membrane skeleton is a network of proteins that helps to maintain the shape of RBCs. It is also important for the deformability of RBCs.

### Carbohydrates in cell membranes

Carbonate residues also are composed as a functional part of the cell membrane of RBCs, which exist in different forms of modifiers on proteins or lipids. Glycoproteins are mainly glycophorins, which are the determinants of blood types as above, and glycosphingolipids also are the antigens that define blood types. The content of sialic acid is quite high in carbonate residues, and it is mainly mannose and galactose as the main stem of carbonate residues, and fucose and sialic acid as the branches.

#### Sialic acid

Sialic acid is the most common negatively charged carbohydrate residue on the RBC membrane. It is present in the form of sialoglycoproteins, such as glycophorins A, B, and C. Periodic acid-Schiff stain-1 (PAS-1) and periodic acid-Schiff stain-2 (PAS-2) are two different forms of glycophorin A that can be distinguished by sodium dodecyl sulfate-polyacrylamide gel electrophoresis (SDS-PAGE). PAS-1 and PAS-2 can interconvert, but their relative proportions change with RBC age. Many researchers have reported that sialic acid, also known as N-acetylneuraminic acid, plays a significant role in the circulation time of RBCs 13. Sialic acid helps to protect RBCs from destruction by the immune system and also helps to maintain the flexibility of the RBC membrane.

#### The structure of sialic acid

**Figure 2:**
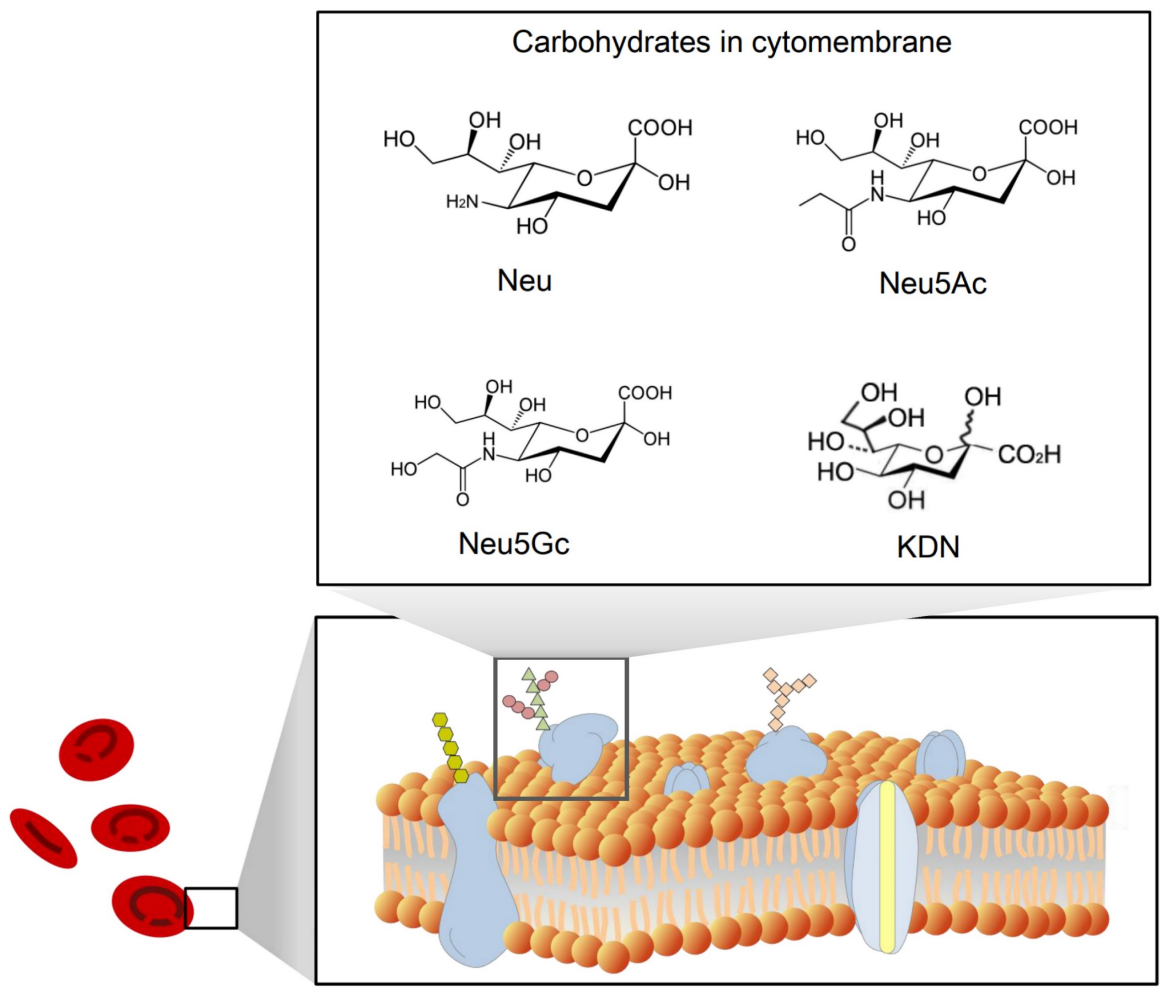
A schematic view of the structure of sialic acid (Neu) 15 and its derivatives Neu5Ac, Neu5Gc, and KDN.

Sialic acid is a monosaccharide with a nine-carbon backbone and is widely expressed on the external surface of RBCs. It is a derivative of neuraminic acid, which can be N- or O-substituted. There are 43 derivatives in the sialic acid family, which can be classified according to their structure and configuration (Figure 2). The most common derivatives in the cell membrane are N-acetylneuraminic acid (Neu5Ac), N-glycolylneuraminic acid (Neu5Gc), 2-keto-3-deoxy-D-glycero-D-galacto-nononic acid (KDN) 14, and N-acetyl-9-O-acetylneuraminic acid (Neu5AcAc).

#### Sialic acid distribution on erythrocyte cell membranes

Sialic acids are components of the cell membrane, typically found at the terminal position of N- and O-linked glycans, as well as on glycosphingolipids expressed at the cell surface. Many secreted glycoproteins are also modified with sialic acid 16. The sialic acid composition of erythrocytes varies among species. For example, Neu5Gc is abundant in many mammals but not in humans 11. Sialic acid together with proteins forms glycoprotein glycophorins (A, B, and C) at the outermost surface of the membrane. Glycophorin A is a protein of 31 kDa with an unknown function in normal erythrocytes. It is the most abundant sialylated glycophorin and contains 51% carbohydrates 6, 17. However, 50% of sialic acids in snake muscle are O-acetylated even after acid hydrolysis.

#### Functions of sialic acid on erythrocyte cell membranes

Sialic acid is a multifunctional molecule that regulates molecular and cellular interactions. It has been shown to play important roles in immunity, homeostasis, inflammation, and antioxidant activity 18, 19. The zeta potential of erythrocytes is primarily determined by the sialic acid on the cell surface, which provides a high negative charge density. This negative charge has an affinity for water molecules, which helps to prevent the erythrocytes from sticking to each other and to the walls of blood vessels 20. Sialic acid also affects the morphology, deformability, and aggregation of erythrocytes. It is also a marker of cell senescence 21. Oxidative stress can lead to the desialylation of erythrocytes 22, which can alter their rheological properties 23. The decrease in sialic acid content in erythrocytes may also affect their lifespan. However, the precise influence of sialic acids on the long circulation times of erythrocytes is still not fully understood.

## Engineered U-RBCs

**Figure 3:**
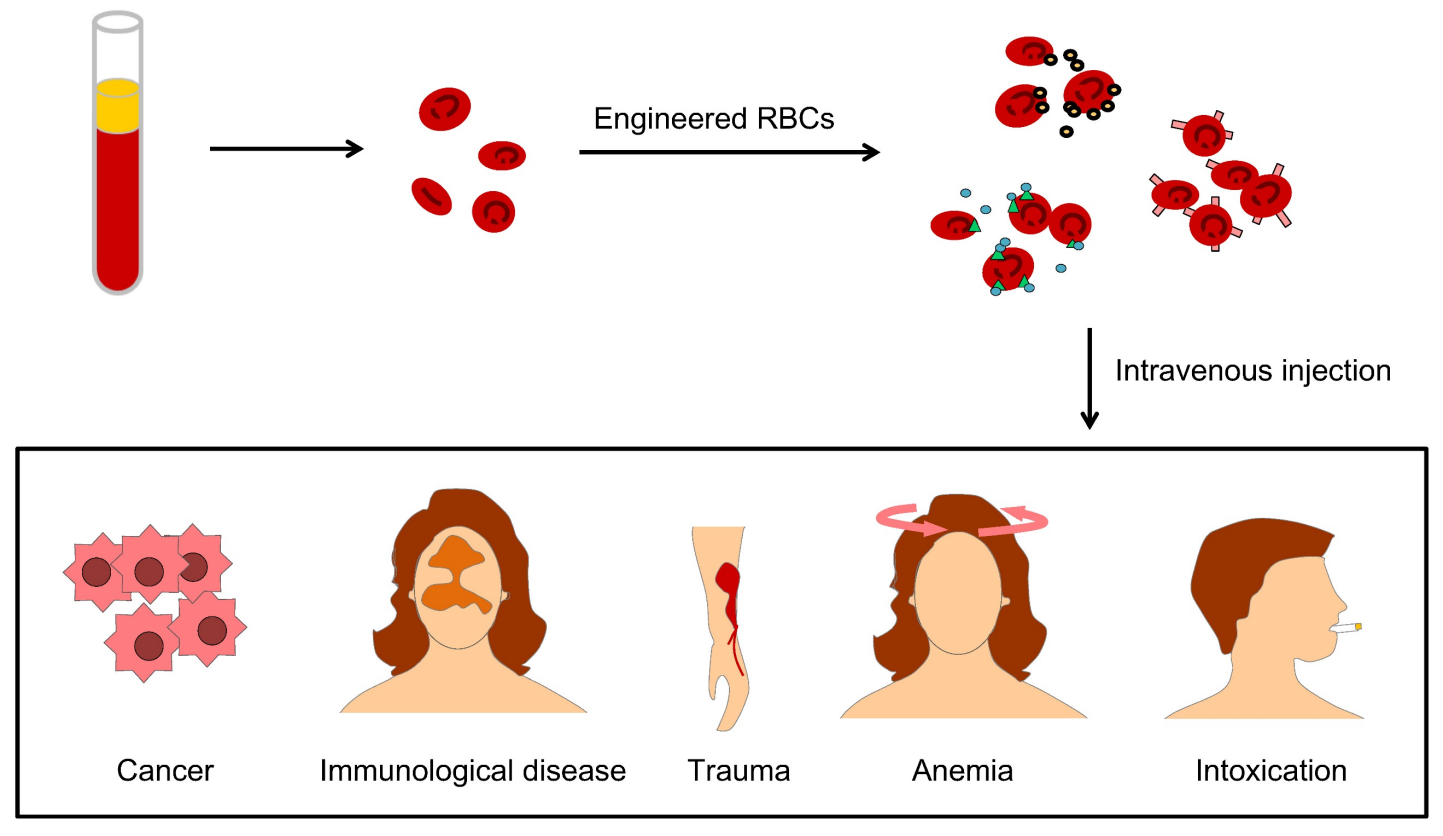
The applications of engineered RBCs for drug delivery include different aspects, such as lack of blood in cancer patients, immunological disease, large physical trauma, kinds of anemia, and clearance of blood toxication.

Engineering U-RBCs is a potential solution to the global blood shortage, and RBC engineering particles also override many obstacles in drug delivery, therapeutic targeting, and biological imaging due to their excellent physiochemical and biological characteristics of longer circulation time, targeting-mediated, good biocompatibility, and attenuated immune response 24. These strengths benefit many clinical problems, such as lack of blood in cancer patients, immunological disease, large physical trauma, kinds of anemia and remove toxins from the blood (Figure 3).

U-RBCs, such as O-negative RBCs, do not have AB antigen groups and can be transfused to any recipient regardless of the kind of preexisting AB antibodies. The goal is to develop a safe and effective way to provide blood transfusions to people of all blood types. Therefore, the O-negative group and the RBCs that are modified artificially and functionally assemble into O-negative RBCs, negative for all of these antigens, and are regarded as U-RBCs, for example, enzymatically converted group O cells and stealth cells 25. There are several ways to produce U-RBCs. One way is to remove the blood group antigens from the surface of RBCs by specific enzymes. Another way is to mask the blood group antigens using biocompatible molecules, such as PEGylation or dopamine. A third approach is to generate U-RBCs *in vitro* from stem cells. This involves genetically modifying the stem cells to remove the genes that code for the blood group antigens. Finally, it is also possible to create artificial U-RBCs from materials science.

### Enzymatic approaches for the generation of U-RBCs

Blood group incompatibility is a major obstacle to blood transfusions for patients with massive blood loss. The enzymatic approach is to solve this problem with biochemical methods.

L. Lögdberg *et al.* (2005, 2011) worked on the chromosomal locations and cloning of human blood groups, and until 2010, They have identified 33 blood group systems that represent more than 300 antigens 26, 27. The ABO blood type system was the first blood group system to be recognized in 1900. The antigens of ABO blood type system is a repeating Gal-GlcNAc disaccharide unit, which is the major polylactosamine chain structure in human RBCs, and this unit can be attached to the membrane either as a glycolipid or as an N-linked glycoprotein 28. The specific chain structures are listed below (Table 1).

The ABO blood group system is the most important blood group system in the clinic. Each blood group has a corresponding antibody in the plasma, except for type O blood. This is why type O blood is considered the universal donor blood type. To date, homotypic principle of blood transfusion is still the main principle. The human immunodominant on the type A RBC membrane is N-acetylgalactosamine (GalNAc), and galactose (Gal) is on the membrane of type B RBCs. Type O blood is the oldest blood, which has neither A nor B type antigens, and there are A and B antibodies in the plasma. Type AB is newly oriented, and both type A and B antigens are expressed on the external surface of the RBC membrane together. Genetically, the A and B types of blood groups were inherited and transformed from type O.

Enzyme-mediated generation of U-RBCs is to remove blood-type antigens (GalNAc and Gal), which means that an N-acetylgalactosamine transferase or a galactose transferase can remove the A and B epitopes, respectively.

#### Basic principles of the enzymatic approach

Antigens are molecules that can be recognized by the immune system. They can be proteins, glycoproteins, or glycolipids. The terminal residue of an antigen is the outermost sugar molecule in its carbohydrate chain, which determines the difference between type A and type B blood. To remove the terminal residue of the antigen or to remove the component on the extracellular layers of the membrane surface, the efficiency of the glycosidases and the proper procedures are essential, because cell viability and compatibility are key criteria to meet. If the glycosidases are not efficient, or if the procedures are not proper, the antigen may not be completely removed from the cell surface, and would not be accepted by the recipient's immune system.

#### Removal of the terminal residue of the type B antigen

**Figure 5:**
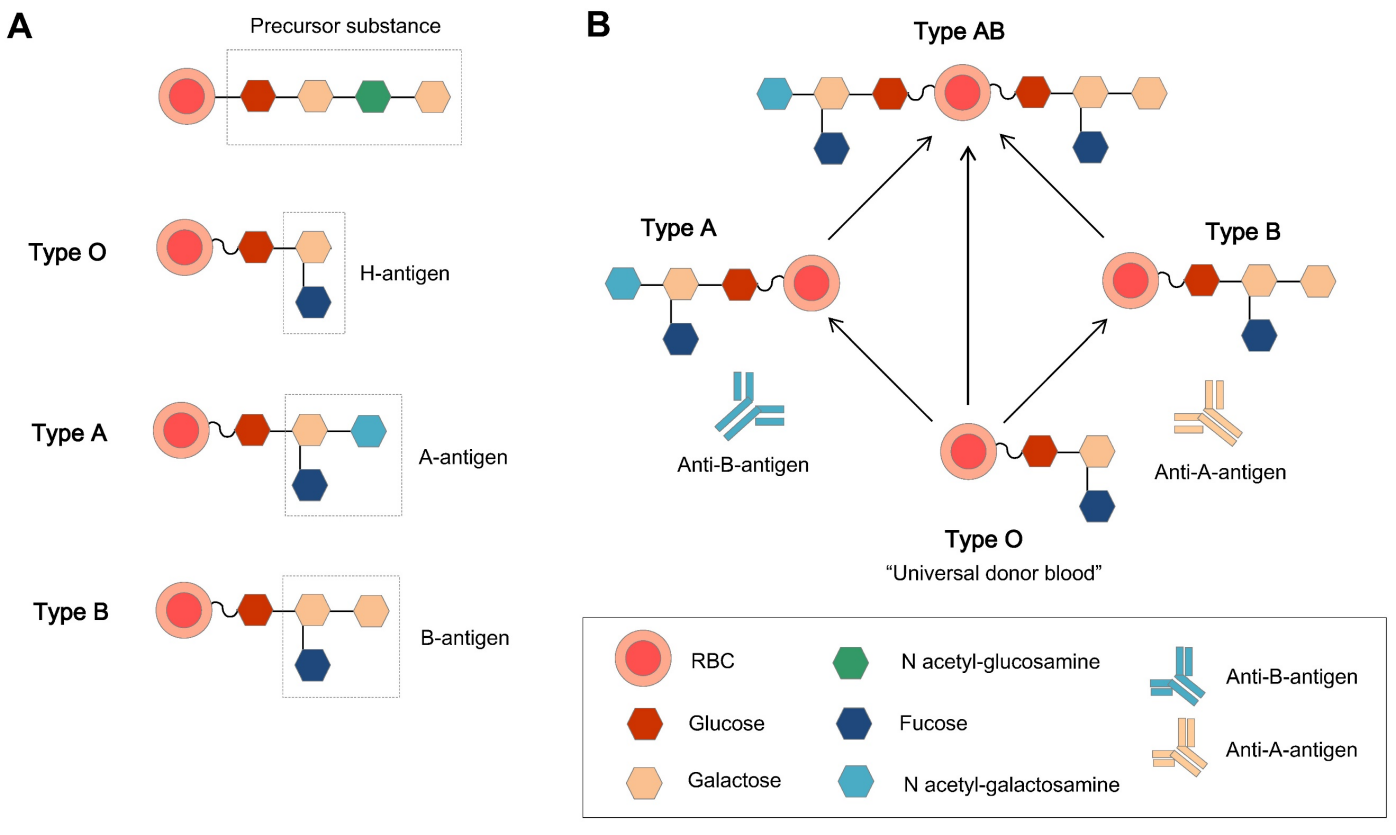
The relations of H, A, and B antigens and the basic structure of the ABO blood group. (A) The original ABO blood group is a precursor substance composed of glucose, galactose, and N-acetyl-galactosamine, and the H antigen (the precursor H oligosaccharide antigen) is the basic unit in the A and B antigens 40. (B) The antigens and related antibodies of the ABO blood group, which make the type O group the natural universal donor.

Enzyme-converted group O (ECO) are RBCs that have been converted to have the same blood group antigens as type O RBCs. This is done by using enzymes to remove the terminal residues of the A and B antigens. Enzymes to cleave the terminal residue of the B antigen have been successfully used in humans, but it is slightly more difficult to remove the terminal residue of the A antigen. To convert group B RBCs to group O RBCs, the Gal residues must be removed. The enzyme for the removal of the terminal residue of the B antigen is alpha-galactosidase 31. The relationship between the ABO group antigens is shown in Figure 5. Most enzymes used in experiments are produced in bacteria for efficiency and economy. However, in 1960, Zarnitz et al. used a-galactosidase from green coffee beans to remove the B and BP1 (nondialyzable residue from B cells) substance epitopes 32. Eleven years later, Yatziv et al. (1971) used α-galactosidase extracted from coffee beans to treat type B RBCs and observed the disappearance of type B blood characteristics 33.

#### Removal of the terminal residue of the type A antigen

α-N-acetylgalactosaminidase produced by *Clostridium perfringens* has been used to convert type A red cells 34, but the antigens cannot be completely eliminated. This is because the A group has two subtypes, A1 and A2 35, and it is more difficult to remove the A antigen on the RBC surface than the B antigen. Additionally, the sources of enzymes are quite limited.

Rahfeld P *et al.* (2019) identified an enzyme pair from the obligate anaerobe *Flavonifractor plautii* among the human gut microbiome that can efficiently convert types A and B red cells to type O of the same rhesus 36. This enzyme pair is more effective than α-N-acetylgalactosaminidase and is more readily available.

New sources of enzymes need to be found to produce ECO RBCs from A1 and A2 RBCs. These enzymes should not interact with monoclonal anti-A antibodies. However, the problems with polyclonal human antibodies (i.e., cross-matching) are still in need of researchers to solve them.

#### Overall limitations of the enzymatic approach to generate U-RBCs

The enzymatic approach to generating U-RBCs has several limitations. One limitation is the need for proper enzymes. The enzymes used in this approach are typically extracted from bacteria 37 or coffee beans 31, and they require specific pH (pH 5.5) and temperature conditions to work properly. These conditions are often not compatible with the membrane osmotic properties of RBCs, which can damage the cells and make them unsuitable for clinical use. Antigens on RBCs determine their activities, such as type A, B, and O. The ABO blood type is the most well-known blood type and is the primary determinant for blood transfusions.

The major human alloantigen systems involve the ABO, Rhesus (Rh), MNS, Lutheran, and Kell blood groups. The Rh blood group system is a clinically significant human blood group polymorphism in alloimmunization and includes more than 50 different serologic specificities 38, making this system particularly important and challenging for biomedical scientists and clinicians. Within the Rh blood group, the D antigen is the most immunogenic and clinically important epitope 6.

The enzymatic removal of the specific antigens of groups A and B from RBCs is a feasible strategy to generate type O cells. However, this technology does not successfully produce U-RBCs. Type O-negative RBCs are considered U-RBCs for emergency conditions.

U-RBCs should not only lack the reactivity of the A or B antigens of RBCs but also lack the reactivity of the D antigen, which is highly immunogenic. Unlike the A and B antigens, the D antigen is a protein-based antigen and is closely associated with the RBC membrane 39. Therefore, it is not possible to excise the immunogenic epitopes of the D antigen from the RBC membrane. Thus, type O-negative RBCs cannot be generated from type A-positive or B-positive RBCs via an enzymatic approach.

### Masking of the blood group antigens from the cognate antibodies

**Figure 6:**
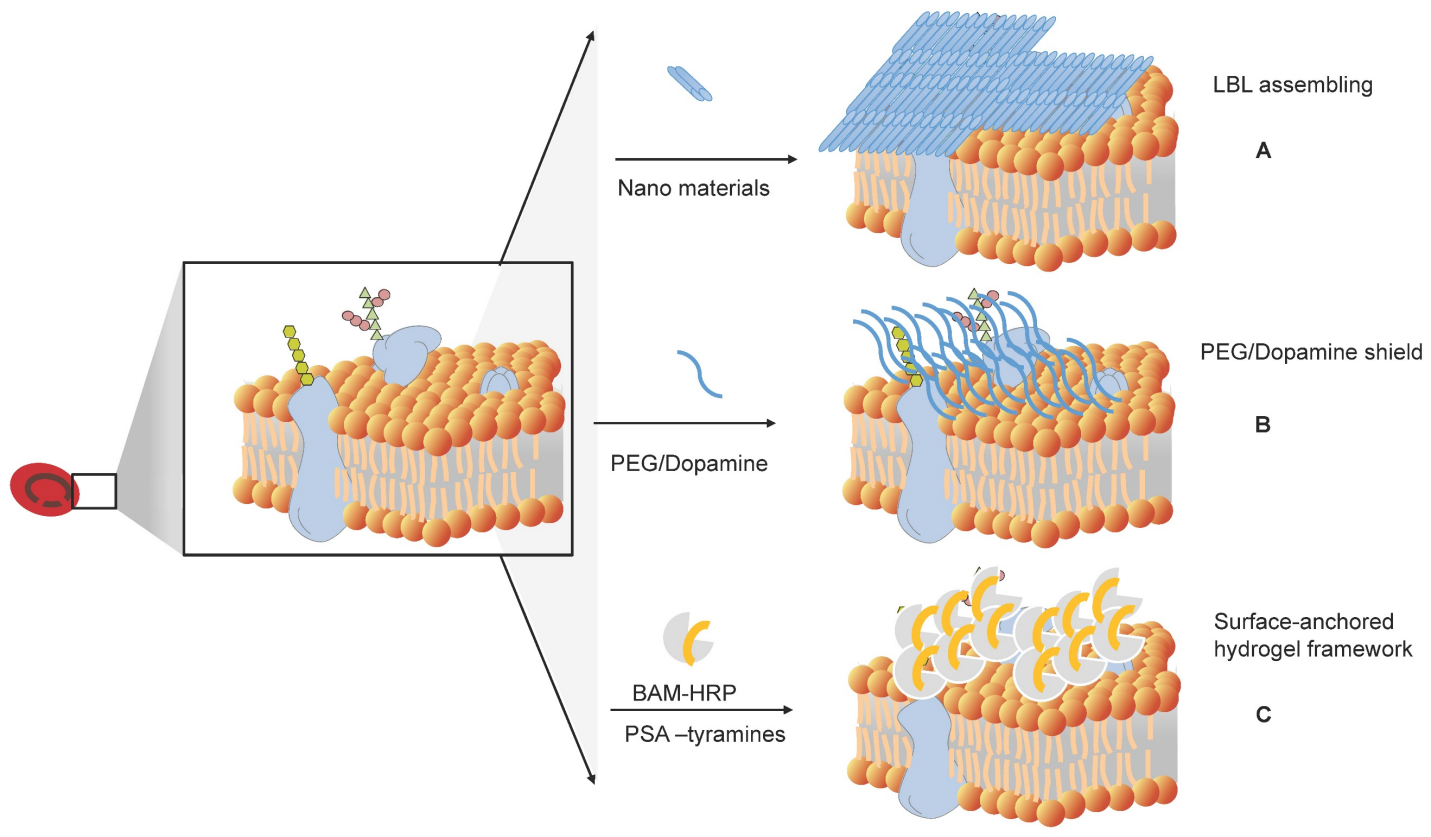
How to shield the original antigens and produce universal RBCs. (A) Layer-by-layer (LbL) assembly to mask antigens on RBCs. (B) Shielding of the original antigens of RBCs by modifying antigens on the membrane surface of RBCs, such as PEG and dopamine. (C) Surface-anchored hydrogel framework for generating RhD epitope stealth RBCs.

Antibodies in plasma react with antigens on the RBC membrane to cause RBC aggregation. An alternative way to prevent this reaction is to block the antigens. Several methods have been developed to produce universal or stealth cells (Figure 6).

#### Antigen masking of RBCs by layer-by-layer (LbL) assembly

Sequential deposition of poly(ethylene glycol) (PEG) on RBCs is a practical way to create a layer-by-layer (LbL) assembly on the membranes. LbL PEGylated RBCs have a prolonged circulation time compared to both poly(lactic-co-glycolic acid) (PLGA) nanoparticles and the corresponding PEGylated nanoparticles 41.

Sania Mansouri et al. (2009, 2011) built a hybrid LbL system with different sets of polyelectrolytes: chitosan-graft-phosphorylcholine (CH-PC) and sodium hyaluronate (HA), and poly-(l-lysine)-graft-poly(ethylene glycol) (PLL-PEG) and alginate (AL). They used these polyelectrolytes to create two types of shelters on the RBC membrane. This LbL system was able to protect RBCs from hemolysis and inhibit recognition by the anti-A antibody 42.

They further tested different polyelectrolytes and found that the optimized shell was composed of four bilayers of alginate (AL) and chitosan-graft-phosphorylcholine (CH-PC) surrounded by two bilayers of alginate (AL) and poly-l-lysine-graft-polyethylene glycol (PLL-PEG) 43.

#### PEGylation of RBCs by covalent conjugation

PEGylation is a good way to mask blood group antigens from cognate antibodies. The molecules generally used are polyethylene glycol (PEG) and its derivatives. PEG is a nonionic, water-soluble, amphiphilic polyether with good biocompatibility. Its molecular weights (MW) vary from 100 to 8,000,000 Da, and it can be configured as a linear PEG, branched PEG (bPEG), or star PEG (sPEG). mPEG is a linear-form PEG capped with a methyl (Me) group.

The virtue of PEG solubility in water is due to the bonding of each ethylene oxide unit with three H_2_O molecules. Coupling reactions occur in the terminal hydroxyl group 44. Commonly, the PEG linkers that form RBCs are cyanuric chloride (CN)-PEG, N-hydroxy succinimidyl (SP)-PEG, and benzotriazole (BTC)-PEG. S. Hashemi-Najafabadi et al. (2006) studied the optimum PEGylation conditions and found that pH 8.7 and 14 °C were the most suitable covalent conditions for PEG of 5 kDa to human RBCs 45.

Cyanuric chloride-PEG, also known as PEG-dichloro triazine (PEG-DCT), is the product of the reaction of trichloro triazine and MeO-PEG-OH (MW 5,000 Da) 46. PEG-DCT was used to couple with the RBC membrane surface, and the PEG-coupled RBCs did not react with plasma antibodies. However, this method is not stable in lactic acidosis. Moore and colleagues studied the stability of engineered RBCs with maleimide-PEGylation and cyanuric chloride-PEGylation masking by agglutination tests with anti-D sera. They found that maleimide-PEG-RBCs showed more stability than cyanuric chloride-PEGylation under multiple conditions. They also found that critical concentrations of lactic acid caused cyanuric chloride-PEG-RBC dePEGylation 47.

Succinimidyl derivatives of PEG (SP-PEG) are similar to a decoration of PEG by succinimidyl. Succinimide derivatives have succinimide radicals or functional groups, which can help PEG gain circulation time. Extension arm facilitated PEGylation is an approach using succinimidyl derivatives of PEG 48. PEGylation occurs by adding an extension arm on the amino groups of RBC membrane proteins. The positive charge of the extension arm successfully covers the A and D antigens of erythrocytes, and the RBCs get a mask to be immune from blood type antibodies in the serum. This method can keep the oxygen affinity of RBCs unharmed. In this approach, Mal‐Phe‐PEG‐5,000 and Mal‐Phe‐PEG‐20,000 chains are needed to mask the A and B antigens. These two molecules in combination can inhibit the agglutination of RBCs with anti-A or anti-B antibodies 49.

The stability of antigen-masked RBCs must be validated before transfusion into blood recipients. Many studies on the masking method to produce U-RBCs have shown its stability and ability to shield RBCs from immune components, and its potential for clinical use. However, Garratty et al. (2008) found that PEG is immunogenic in animals and humans, and the occurrence of PEG antibodies can shorten the circulation time of masked RBCs or proteins 44.

Antigenically shielded U-RBCs by polydopamine (PDA)-based cell surface engineering

Dopamine is a neurotransmitter and plays a key role in the neuronal system. In the meantime, it is a new star molecule that is widely used in allosteric molecular chemistry. It can tightly bond on solid surfaces and has good biocompatibility. In the bioengineering field, polydopamine-coated hemoglobin can work as an oxygen carrier. PDA is widely used as a carrier in polymeric nanodrugs after PDA-based surface modification 50. Wang, B., *et al.* (2014) used PDA-based cell surface engineering 51 to produce U-RBCs with good membrane permeability, fluidity, and high oxygen capacity. The PDA coating shielded the antigenic epitopes on the RBC surface, making them compatible with all blood types. *In vivo* studies showed that the PDA-engineered RBCs had a long circulation time and were able to deliver oxygen effectively. Polydopamine (PDA) can also be used as an antioxidant enzyme. Liu et al. (2018) encapsulated a complex of enzyme-mimicking PDA carrying hemoglobin into the membrane of RBCs. These man-made RBCs are an oxygen-self-supplied platform that can be used to overcome the hypoxia-mediated resistance of tumors to photodynamic therapy 52.

#### Surface-anchored Hydrogel Framework for Generating RhD-epitope Stealth RBCs

Rhesus D (RhD) is another important blood type determinant 53. RhD-positive blood is common in humans, which can make it difficult to find RhD-negative blood for transfusion. Zhao et al. (2020) developed a surface-anchored hydrogel framework to shield the antigens on the surface of RBCs with polysialic acid-tyramine. This completely obstructed the RhD antigens on the cell surface, and the surface-anchored framework provides an efficient method for generating U-RBCs for blood transfusion 54.

### *Ex vivo* synthesis of U-RBCs

Blood transfusion is a cell therapy that is essential for many medical procedures and treatments. However, blood supply is not always available, and the storage of blood can be challenging due to the rapid deterioration of blood cells. *Ex vivo* synthesis of U-RBCs could address these challenges by providing a consistent and reliable source of blood.

#### General principles of the approach

Hematopoietic stem cells (CD34^+^ cells) in blood, bone marrow, or cord blood can generate erythroid cells. Luc Douay et al. (2007) developed a protocol for the expansion and differentiation of progenitors into mature RBCs *in vivo*. This approach is highly effective, but it is also expensive 55.

The Malik P. group (1998) found that adding specific combinations of growth factors in the right order to culture hematopoietic stem/progenitor cells (HSPCs) can induce erythropoiesis 56. Neildez-Nguyen et al. (2002) cultured HSPCs (isolated from cord blood, or CB) by adding cytokines Flt3 ligand (cytokine Fms-like tyrosine kinase 3 ligand) 57, thrombopoietin, stem cell factor, erythropoietin, and insulin-like growth factor I into a culture medium in a specific order to produce RBCs from induced pluripotent stem cells (iPSCs), which are similar to embryonic stem cells (ESCs).

In addition to human hematopoietic/progenitor stem cells, human embryonic stem cells and induced pluripotent stem cells have already been used to generate functional RBCs. Blood, bone marrow, and cord blood are all possible sources of cells. Akihito Fujimi et al. (2008) obtained the large-scale generation of human RBCs from cord blood CD34^+^ cells by *ex vivo* co-culturing with macrophages 58. RBCs generated from ESCs require the differentiation of ESCs, but the protocol for generating RBCs has limited efficacy 59.

Universal blood group-based iPSCs offer the benefits of removing blood groups, as well as HLA-ABC-deficient platelets and myeloid cells 60. This makes it possible to produce functional RBCs *in vitro* on a large scale 61.

#### Functional properties of the *ex vivo* cultured RBCs

*Ex vivo* cultured RBCs can provide a large number of uncontaminated, mature RBCs for clinical and research use. Peptides and molecules that define erythropoiesis are important in the process of *ex vivo* cultured RBCs from stem cells. Enucleation is induced to promote the maturation of RBCs.

Some functional properties between normal, original RBCs and ex vivo cultured RBCs have been measured. The levels of glucose-6-phosphate dehydrogenase and pyruvate kinase are comparable to those of native RBCs, so ATP production and reduced glutathione can be provided by *ex vivo* cultured RBCs. Hemoglobin function and oxygen equilibrium are very close to those of native RBCs 55.

David J. Anstee et al. (2012) reviewed the RBCs generated from hematopoietic stem cells *ex vivo* and discussed the quality of these cells for transfusion. They concluded that all the functions of universal cells made by *ex vivo* hematopoietic stem cell culture meet the criteria 62, although more time is needed to meet the great shortage of transfusions.

Sabine Kupzig *et al.* (2017) compared the lifetime of *ex vivo*-generated RBCs and donor red cells using an *in vivo* model and showed that the former survived longer than the latter and that *ex vivo*-generated red cells cultured from human reticulocytes more closely met the demand of clinical transfusion.

#### Bioengineering of U-RBCs

The bioengineering of U-RBCs involves changing the antigens on the surface of RBCs using biological methods.

Sho-ichi Hirose et al. (2013) found that transducing c-MYC and BCL-XL genes into multipotent hematopoietic progenitor cells can overexpress these genes and induce the immortalization of erythrocyte progenitor cells 63. This allows for the large-scale production of RBCs. Katie E. Glen et al. (2013) reported a method for the production of erythrocytes from directly isolated or Delta1 Notch ligand-expanded CD34^+^ hematopoietic progenitor cells 64. This method has a high potential for manufacturing production. Jan Frayne et al. (2019) used combinatorial gene targeting to create individual and multiple blood group knockout sublines, such as ABO [Bombay], Rh [Rhnull], Kell [K0], Duffy [Fynull], and GPB [S-s-U-] 65.

With the development of culture erythroid progenitor cell lines from a variety of sources, such as adult peripheral blood mononuclear cells and even some common cell lines 66, and the reduction in the cost of culturing *in vitro* RBCs 67, it will be much more convenient to expand the bioengineering capacity for U-RBCs.

### Artificial RBCs

Artificial RBCs are man-made membranes that encapsulate hemoglobin (Hb) and enzymes to make them resemble natural RBCs. Artificial RBCs can circulate for a longer time than natural RBCs and have similar properties, such as oxygen-carrying, flexible deformation, and semi-permeable membranes. The development of artificial RBCs is a necessity 68. Hemoglobin-based oxygen carriers and perfluorocarbon-based products are some of the successful examples of artificial RBCs. In 2003, Chang, T.M. et al. reported artificial RBCs based on ultrathin polyethylene-glycol-polylactide (PEG-PLA) membrane nanocapsules containing Hb and enzymes. After optimization with polymerized Hb, higher molecular weight, higher concentrations of PEG-PLA, and cross-linking of the newly formed PEG-PLA Hb nanocapsules, the circulation time of Hbs dropped to 1.67 gm/dl after 41.5 hours 69.

Artificial RBCs can also be used for cancer treatment. For example, mitochondria-targeted artificial "nano-RBCs" can be used for amplified synergistic cancer phototherapy by single near-infrared region irradiation 70. They can also relieve tumor hypoxia and enhance cancer radiotherapy with erythrocyte-membrane-enveloped perfluorocarbon 71.

With the development of bioinstrumentation and technologies, the precise structures and functions of RBCs have become more available. All of the improvements in biotechnology have led to the *ex vivo* synthesis of U-RBCs and artificial RBCs.

## Red cell-based advanced drug delivery system

Drug delivery is a major technical challenge in drug development and clinical therapy 72-74. A good drug delivery system should achieve the maximum therapeutic effect with the lowest side effects 75. The drug carrier must protect the delivered cargo's effectiveness and accessibility, as well as load and release the drugs properly. Since the blood circulatory system is present throughout the body, most drugs that are injected into the bloodstream can be transported by blood 76. Additionally, RBCs have a fascinating size and shape, an almost empty structure inside, and flexible deformation, making them the most promising drug delivery carriers 77. Chen, S. et al. (2021) synthesized the nanomedicine poly(2-(N-oxide-N,N-diethylamino)ethyl methacrylate) (OPDEA), which reversibly attaches to RBCs and prolongs the circulation time of the anticancer drug SN38 78.

**Figure 7:**
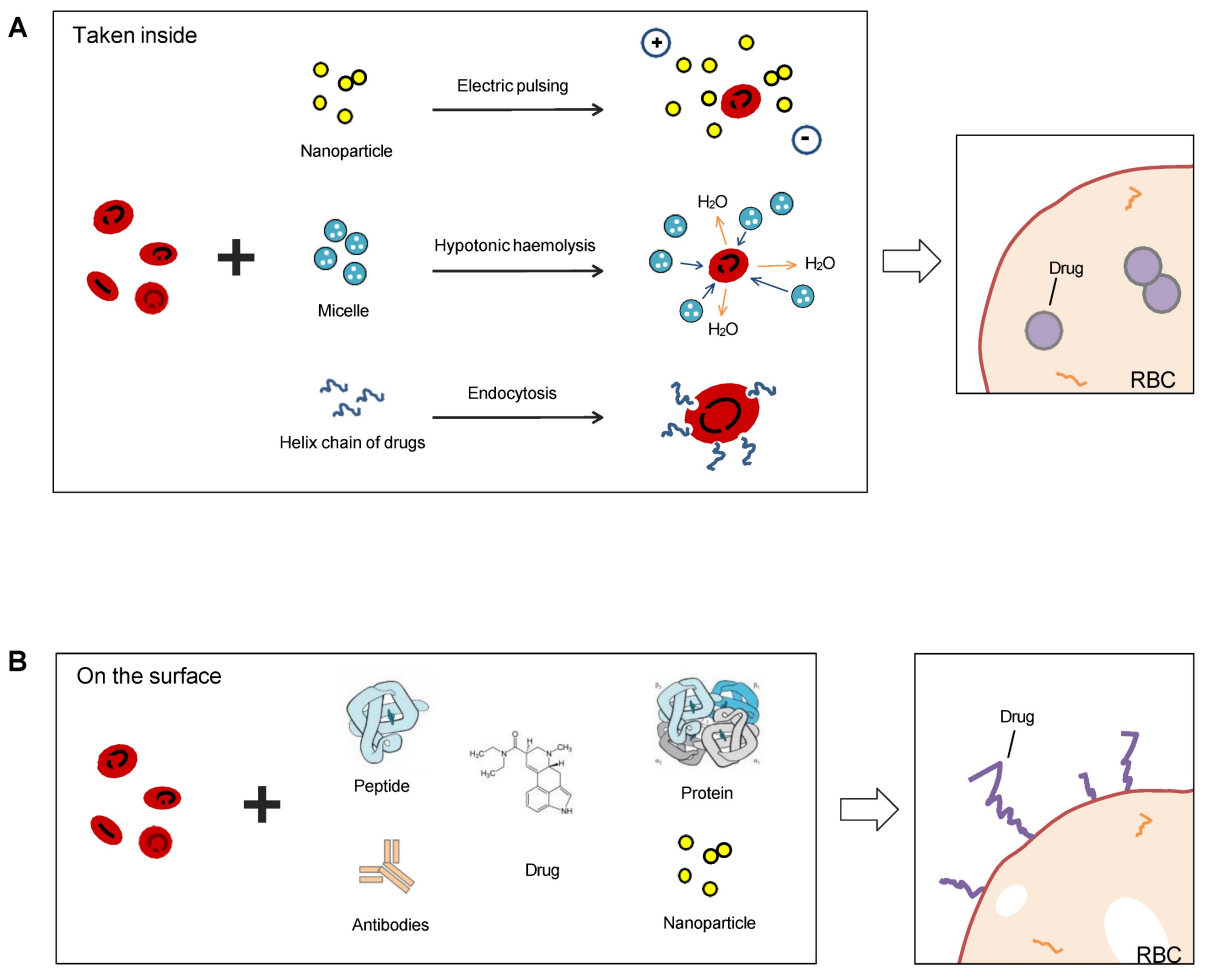
Sketch illumination of RBCs as a drug delivery platform precedent hitchhiking. RBCs can be used for drug delivery in two ways: taking drugs inside or binding on the surface. Electric pulsing, hypotonic hemolysis, and endocytosis are common means to invade the bilayer membrane of RBCs and absorb nanoparticles or other forms of drugs in (A), and another means is linked antibodies, peptides, proteins, or chemicals directly on the surface (B).

Most common methods to use RBCs as drug carriers are encapsulating the therapeutics into the cavity of the membrane or coupling them on the membrane surface (Figure 7), and RBC-hitchhiking is a new drug-loading technique (RBCH) applied nanocarriers noncovalently onto RBCs, it relies on physical forces such as van der Waals forces, hydrogen bonding, and electrostatic interactions to couple RBCs and cargoes.

The packaging of drugs or other cargoes into RBCs can be achieved before or after the RBCs mature. One approach is to use stem cell-erythroid precursors to produce long-term systemic proteins that can target specific tissues. Another approach is to couple drugs or other cargoes to the surface proteins or carbohydrate molecules of RBCs. RBCs can also be used as convenient tracers when carrying magnetic particles. In addition, RBC-mimicking synthetic biomaterial particles and erythrocyte membrane-camouflaged polymeric nanoparticles have been developed and used in practice. Carlos H. Villa *et al.* (2017) reviewed the pros and cons of different sources and loading types for drug delivery and pointed out four key factors that determine whether the loading was successful: the optimal loading protocol, the source of the RBCs, the production logistics, and regulatory hurdles 79. We will analyze the different drug delivery techniques separately below.

### Stem cell-derived erythroid cells mediate long-term systemic protein delivery

Erythroid precursors are born from bone marrow, and these cells have nuclei. During the differentiation and maturation of RBCs, they obtain the proper components inside and on the cells. The nuclei are released by exocytosis. Using a genome-editing technique, some scientists can use stably expressed and rationally designed proteins inside RBCs to carry specific drugs. In a mouse model of hemophilia B, Alex H. Chang *et al.* (2008) used gene editing technology to target human factor IX (hFIX) expression in late-stage erythropoiesis. They achieved long-term hFIX secretion (18 months) at levels tenfold higher than those obtained with an original promoter 80. Phetcharat Phanthon* et al.* (2017) used pluripotent stem cell (iPSC)-derived erythroid cells as a platform to enhance β-globin gene expression in thalassemic IVS2-654 by modified U7 snRNA. Erythroblasts differentiated from transduced iPSCs expressed high levels of correctly spliced β-globin mRNA, which could be used to treat beta-thalassemia 81.

### Vascular delivery of drugs encapsulated into RBC carriers

The encapsulation of drugs into RBCs can be achieved by electrical insertion or hypotonic loading. Diverse agents have been encapsulated into RBCs, such as small molecules and therapeutic proteins 82.

There are three main characteristics of encapsulated drugs in the inner volume of RBCs. First, the RBC ghost can encapsulate drug compounds that cannot pass through the plasma membrane. Second, encapsulation also helps to protect the drug from the body's immune system because the drug cargo is separated from the body. Third, the drug-loaded RBCs are only a small fraction, which is important for patients who have a low blood count 83.

The loading efficiency and release rate of different agents are important indices to be measured. Ropars C. and his colleagues (1987, 1996) found that inositol hexakisphosphate encapsulated in RBCs improved the oxygen capacity of RBCs 84. Al-Achi A. and Greenwood R. (1998) investigated the oral administration of human insulin by human RBC encapsulation 85, and most of the projects worked well. Biologicals have also been successfully encapsulated in RBCs for antiviral interventions 86 or gene therapy 87.

Monica Piergiovanni et al. (2020) used fluidic shear stress to make the pores on the membrane of RBCs open for a short time and allowed the drug fluid (FITC-dextran, 40 kDa) to diffuse into the RBCs successfully. This experiment was beneficial to the equipment they mentioned in the study, the computational fluid dynamic model, and the microfluid channels 88.

Other pharmacological effects, such as chemotherapeutic agents in cancer chemotherapy, have also been studied 89. For example, photosensitized erythrocytes loaded with methotrexate were found to release the drug rapidly. Kim et al. (2009) reported a quantitation method for enzyme-linked immunosorbent assay (ELISA)-based assay and a carrier-mediated antisense oligonucleotide delivery system based on erythrocyte ghosts 90. Yew et al. (2013) successfully encapsulated phenylalanine hydroxylase into RBCs and evaluated the sustained enzymatic activity in the circulation 91. This proves that enzyme-loaded RBCs can metabolize amino acids in the bloodstream and can be used to treat disorders of amino acid metabolism.

However, there are still some concerns about encapsulation. For example, the encapsulation procedure may damage the inner membrane or surface structure of RBCs, or the biocompatibility may decrease, leading to phagocytosis along the delivery route. Hypotonic preswelling is one of the methods to achieve intact RBC coupling, but it requires the elimination of Cremophor EL (a derivative of ethylene oxide and castor oil) and dehydrated alcohol, which have a high risk of infection and hypersensitivity reactions. In a study by Gamaleldin et al. (2004), 148.8 µg of paclitaxel was loaded per mL of erythrocytes, and as much as 81% of the loaded paclitaxel was released into the plasma in less than 48 hours. However, the coupling method led to lipid and protein oxidation in the erythrocytes 92.

Overall, the encapsulation of drugs into RBCs is a promising approach for drug delivery. However, there are still some challenges that need to be addressed, such as the preservation of the biocompatibility of RBCs, the properties of the drug and the RBCs, and the development of more efficient and controlled release methods.

### Coupling therapeutic drugs to the RBC surface

As mentioned above, the RBC membrane is a complex structure with numerous residues of protein, lipids, and carbohydrate molecules. The biconcave shape of RBCs gives them a surface area that is 1.4 times larger than that of normal spherical cells, which makes them ideal for surface coupling of therapeutic drugs.

The coupling of therapeutic drugs to the RBC surface can be achieved using a variety of methods, including covalent or noncovalent affinity, absorption, adhesion, or attachment. In the early 1980s, scientists used 2-iminobiotin-avidin 93, biotin derivatives, and 125I-streptavidin to couple drugs to amino acids on the membrane surface. Sugar and lipids are also common targets for surface coupling, and many scientists have tried to couple antibody heteropolymers 94 to the RBC surface to eliminate pathogens or toxins in the circulatory system. With the coupling of complement inhibitors to the RBC surface, RBCs can be protected from complement-mediated hemolysis 95, 96.

However, most coupling agents and procedures have the potential to damage the RBC membrane and compromise its biocompatibility. Additionally, the control of drug release kinetics is a challenging problem. Despite these challenges, surface coupling technology has several advantages over other drug delivery methods, such as the ease of extraction and infusion of RBCs and the improved hygiene.

Recent advances in surface coupling technology have made it possible to develop strategies for immune-mediated disease therapy. For example, Novalia Pishesha et al. (2017) developed a method for coupling therapeutic drugs to the RBC surface that was effective in a mouse model of multiple sclerosis and type I diabetes 97.

### RBC-hitchhiking (RBCH) technique

RBC hitchhiking drug delivery system (RBC-H) 24 is a novel drug delivery paradigm that uses transient coupling of nanocarriers to RBCs to deliver drugs to specific organs. This approach has great potential for the treatment of a number of diseases 98. Nanocarriers that hitch a ride on RBCs in a non-covalent manner can travel further and more efficiently throughout the body, which can improve their therapeutic effects 99. This is because RBCs are naturally transported to specific organs, such as the lungs and liver. The RBC-H technique is efficient and safe in delivering drugs to a variety of organs, including the brain, liver, lungs, and kidneys. It is also easier to target the lungs with RBC-H than the spleen, because the shear stress in lung capillaries dislodges particles in the lungs 100, 101. Biodegradable drug nanoparticles assembled onto the surface of RBCs can extend the circulation time and delivery content of the drug 100.

Several studies have demonstrated the potential of RBC-H for drug delivery. For example, Yaning Ding et al. successfully generated RBC-MPSS-CSNPs, which significantly prolonged the circulation time of glucocorticoids, enabling specific delivery to the lung 102. Junyan Li et al. utilized the RBC-hitchhiking strategy to facilitate targeted delivery and alleviate inflammatory syndrome in acute pneumonia 103. Jacob S Brenner et al. reported nanocarriers (NCs) designed based on the RBC-hitchhiking strategy, which significantly increased the absorbance of downstream organs by orders of magnitude. This approach was successful in mice, pigs, and *ex vivo* human lungs without causing RBC or end-organ toxicities 101. Anvay Ukidve et al. leveraged the ability of RBCs to present certain pathogens to antigen-presenting cells (APCs) in the spleen to develop an erythrocyte-driven immune targeting (EDIT) strategy 104. This strategy directly presents antigenic nanoparticles to the spleen, resulting in an improved immune response to the antigen and eliciting a prophylactic effect in a tumor model.

Overall, RBC-H is a promising new drug delivery paradigm with the potential to improve the efficacy and safety of drug therapy for a variety of diseases.

### RBCs as carriers in magnetic particle imaging

Loading superparamagnetic iron oxide (SPIO) nanoparticles onto RBCs is a new method of magnetic resonance imaging (MRI) for long-term monitoring of the retention time and distribution of nanoparticles *in vivo*. SPIO nanoparticles have superparamagnetic properties, which make them suitable for a variety of biomedical applications 105. However, SPIO nanoparticles are rapidly cleared by the reticuloendothelial system (RES) in the liver, spleen, and lymph tissue. This limits their use in biomedical applications 106. With SPIO-loaded RBC technology, the story is quite different. Antonelli et al. (2011) developed a method for loading SPIO nanoparticles onto RBCs. They found that the loading efficiency of the nanoparticles depended on their size, synthesis protocol, coating and/or dispersant agents 107. The hydrodynamic diameter of the nanoparticles should be less than 60 nm to allow them to pass through the pores in the RBC membrane. He loaded several commercial SPIO nanoparticles (SHU 555A, AMI 227, and PMP-50) coated with dextran or carboxydextran and new nanomaterials (Np-1 nanoparticles dispersed in the Disperbyk®-190 agent) into RBCs and compared their loading conditions and efficiencies. The procedure of encapsulating magnetic nanoparticles into RBCs is by hypotonic dialysis and isotonic resealing, followed by reannealing with a few modifications. This method is a promising new approach for using SPIO nanoparticles in biomedical applications. It has the potential to improve the efficacy of MRI imaging and other biomedical therapies.

### RBC-mimicking synthetic particles

Mimicking the biostructural and key functional features of RBCs in synthetic particles is a novel method to combine the advantages of natural and manufactured materials. Biomaterials are widely used in biomedical applications as carriers for drug/protein delivery systems, medical imaging agents, and regenerative medicine 108. Doshi's lab has developed RBC-like particles that mimic the mechanobiological and chemobiological properties of RBCs. These particles offer the engineering control required in synthetic carriers and provide a connection between synthetic materials and biological entities 109. Lippi et al. (2010) demonstrated the use of these RBC-mimicking particles in blood doping. The particles were able to carry a large amount of oxygen to the peripheral tissue 110, which could potentially improve athletic performance.

RBC-mimicking synthetic particles have a number of potential applications in biomedical research and medicine. They can be used as carriers for drug delivery, as contrast agents for MRI, and as cell-free models for studying the biology of RBCs. However, there are still some challenges that need to be addressed before these particles can be used in clinical applications. These challenges include ensuring the biocompatibility of the particles, developing methods for mass production, and optimizing the loading capacity of the particles. Despite these challenges, RBC-mimicking synthetic particles are a promising new class of materials with the potential to revolutionize biomedical research and medicine.

### Erythrocyte membrane-camouflaged polymeric nanoparticles as a biomimetic delivery platform

Coating biodegradable polymeric nanoparticles with natural erythrocyte membranes is a method to combine the advantages of natural complexity and manufacturing efficiency. Natural erythrocyte membranes can help nanoparticles disguise and evade the immune system 111. RBC-derived extracellular vesicles (EVs) and microparticles (MPs) are naturally produced in both normal physiological and diseased states. RBC-derived EVs are promising delivery vehicles for therapeutic agents because they are easy to source, safe, and versatile 112. RBC-derived MPs are a fundamental biological process of cell aging, cell survival, and cell clearance 113. Erythrocyte nanovesicles 114 are novel transporters that can modify the functions of target cells and participate in the physiological and pathological processes of modified targets 115. The drawback of low molecular weight (LWM) chitosan nanoparticles applied in intravascular drug delivery systems is clearance by the reticuloendothelial system. A multifunctional drug carrier composed of LWM chitosan nanoparticles and erythrocytes made by Fan W. et al. (2012) shows the compatibility of the hybrid particles 116.

**Figure 8:**
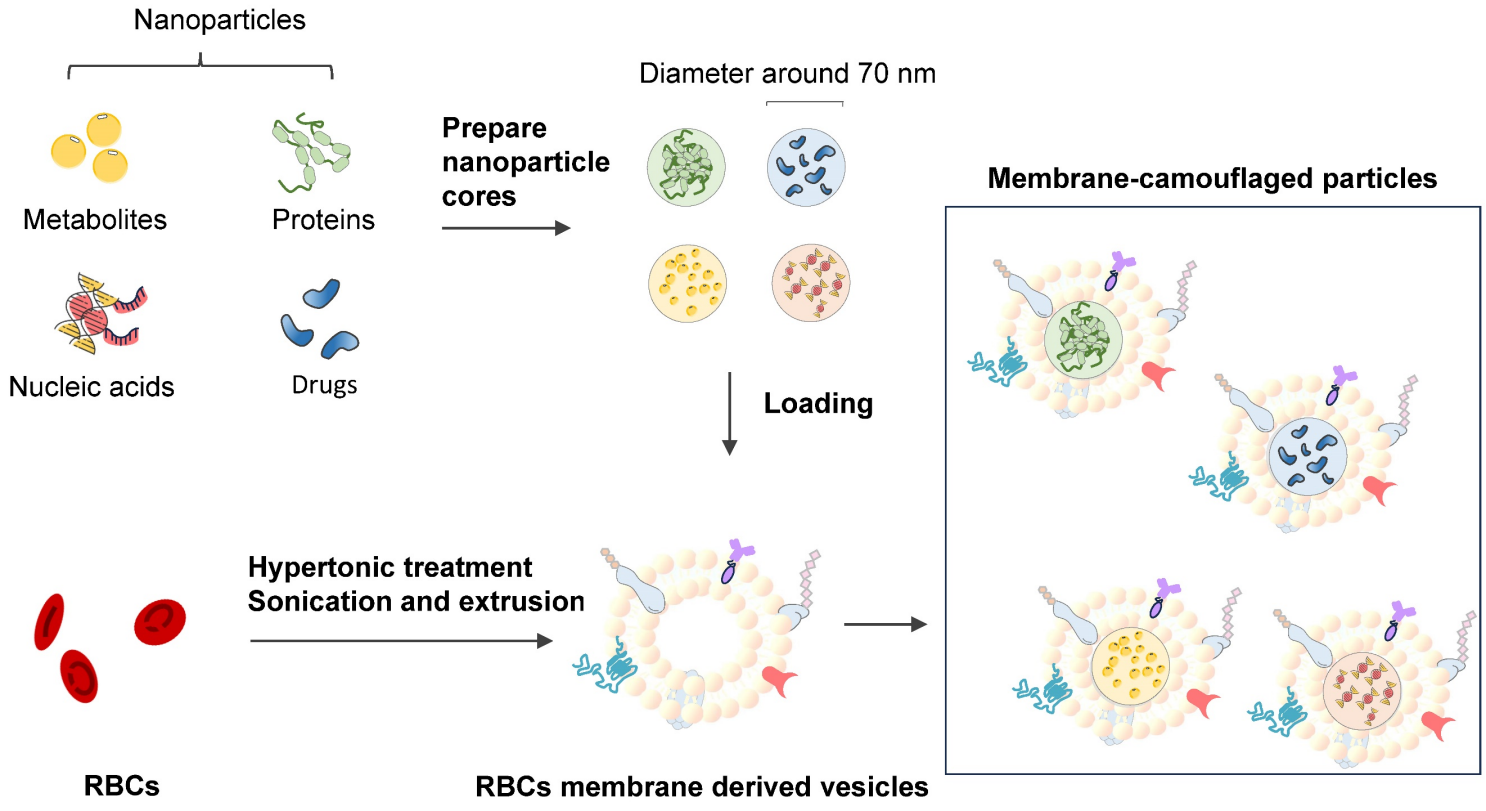
RBC mimicking membranes by part of the RBC components and exhibition of erythrocyte membrane-camouflaged polymeric nanoparticles.

Lai P. et al. (2015) successfully constructed biomimetic stem cell membrane-camouflaged SPIO nanoparticles for theragnostic applications and analyzed the magnetic hyperthermia effect for inducing cancer cell death 118. Nanoerythrosomes (NERs) are nanoengineered erythrocyte ghosts. One kind of drug for pulmonary arterial hypertension, Fasudil, was encapsulated in NERs by Nilesh Gupta and resulted in a 6-8-fold increase in the half-life of Fasudil 119. Zhang et al. (2011) developed a new drug delivery platform that engrafts nanoscale RBC membrane-derived vesicles with polymeric nanoparticles made from polylactic-coglycolic acid (PLGA), an FDA-approved polymer 117 (Figure 8). Rao et al. (2015) utilized the RBC membrane as a biomimetic nanocoating material and prolonged the circulation time of Fe_3_O_4_@RBC nanoparticles due to the lack of elicitation of immune responses at either the cellular level or the humoral level 120. This method could be exploited to reduce accelerated blood clearance by a second injection of PEG modification particles. Cao et al. (2019) used the membrane of RBCs to wrap bacteria and get the bacteria through unavoidable side effects and low treatment efficacies in therapy 121.

### Cell surface coated of RBCs for blood cleaning

Small molecule toxicants can easily accumulate in blood, such as metal ions and small- and medium-molecular-weight organic substances. These toxicants can be absorbed from the *in vitro* environment as well as *in vivo* disease-damaged tissues. Removing toxicants from the blood is of great significance, as sepsis and septic toxic shock can cause more deaths than heart disease.

For decades, erythrocytes have been investigated as a carrier of specific enzymatic antidotes against chemical intoxicants. These enzymes include rhodanase for cleaning cyanide, phosphotriesterase for cleaning paraoxon, acetaldehyde dehydrogenase and alcohol oxidase for cleaning ethanol, formate dehydrogenase and δ-aminolevulinic acid dehydratase for cleaning methanol, and hydrogenase for cleaning hydrogen gas. Most of these erythrocyte-mediated enzymes have been tested in mouse models. However, this approach has limitations, as the metabolic capacity of the erythrocyte-mediated enzyme is restricted to the vascular compartment and monocyte-macrophage system 122.

A new approach to the design of next-generation nanobiomaterials is to precisely engineer their physical and chemical properties with biomimicry. This can create nanoparticles that can circulate through the blood for extended periods of time, act as detoxification devices, and improve survival in a mouse model of sepsis. For example, Elana Ben-Akiva designed biodegradable polymeric nanoparticles coated with RBC membranes for detoxification agents. These red cell membrane-coated nanoparticles enhanced the detoxification rate and decreased the death rate in a mouse model of sepsis 123. Increased phospholipase A2 (PLA2) activity is related to many diseases in pathological conditions, such as autoimmune disorders and cancer 124. Zhang Q. et al. (2020) used a 'lure and kill' strategy to modify RBC membranes with melittin and oleyloxyethyl phosphorylcholine (OOPC) as coverage for nanoparticles. These two key components are melittin for the "lure" and OOPC for "killing" PLA2. The lure and kill nanoparticles can eliminate the toxicity of free PLA2. The mouse model test showed that the lure and kill nanoparticles inhibited hemolysis and conferred a significant survival benefit 125. Li C. et al. (2020) developed a Janus dendrimer amphiphile (JDA) molecule bridging to load the antidote WP6 as a supramolecular hunter and anchored the hunter on RBC membranes. In this way, the toxicant paraquat could be efficiently removed from blood, both *in vitro* and *in vivo* 126.

Overall, blood cell-related DDSs have the potential to be a promising new approach to drug delivery. However, there are a number of challenges that need to be addressed before these DDSs can be widely used 127. More research is needed to improve the accuracy and precision of drug release, reduce the costs and time of production, and better understand the underlying mechanisms of blood cell behaviors.

## RBC-inspired cancer immunotherapy

Cancer immunotherapy is a promising new approach to cancer treatment that uses the body's own immune system to fight cancer cells. RBCs play an important role in cancer immunotherapy, as they can be used to deliver drugs, genes, and other immune-stimulating agents to cancer cells. As immune engineering springs up to produce drugs with biomaterials for enhanced cancer immunotherapy 129, the delivery of these complicated drugs 130 plays a great role in treatment efficacy.

### Progress of drug-loaded RBCs in immunotherapy

RBCs have several advantages for use in cancer immunotherapy. They are abundant, easy to obtain, and relatively inexpensive. Drug-loaded RBCs are also well-tolerated by the body and have a long circulation time, which allows them to deliver drugs and other agents to cancer cells over an extended period of time. Instead of labor- and fund-consuming antigen-loaded monocyte-derived DCs, antigen-loaded RBC-derived DC systems have advantages in their abundant content, biocompatibility, and circulation time. RBCs were used as artificial antigen-presenting cells 128 by Sun X.* et al.* (2017), and successfully stimulated T cells and the following immune response. Dr. Liu Z.'s group used DNA-assisted bottom-up self-assembly to precisely control both the lateral and vertical distributions of T cell activation ligands on the membrane of RBCs 129. This application mimics natural antigen-presenting cells, engineers the cell membrane interface, and tunes cell-cell interactions, and it is promising for use in immunotherapy.

There are several ways to use RBCs in cancer immunotherapy. One way is to load RBCs with drugs or genes that can kill cancer cells. Another way is to engineer RBCs to express immune-stimulating molecules that can activate the body's immune system against cancer cells. A review from Anderson HL demonstrated the immunological function of RBCs, and summarized the humoral and cell-mediated immunity by binding and scavenging various inflammatory molecules to the surface of erythrocytes, involving in chemokines, nucleic acids, and pathogens 130. Another review by Elisabeth Karsten illustrated RBCs' roles in cytokine signaling and in modulating the activity of immune cells 131. Currently, there are several technical platforms to develop new formulations of drugs based on RBCs.

RBC-OVA system: This system uses RBCs to deliver antigens to dendritic cells. Dendritic cells are immune cells that help to activate the body's immune system against cancer cells. RBCs loaded with ovalbumin (RBC-OVA) were used as antigen carriers to target dendritic cells (DCs) 131 by Alice Banz et al. (2010), and the antigen was released by macrophage phagocytosis. Then, the OVA-specific T-cell response was activated to lyse OVA-binding cells. The antigen entrapped by RBCs can be effective for as long as 30 days. Alice Banz in another study 132 tested the utility of this delivery system in two tumor mouse models, using the E.G7-OVA and the B16F10 tumor cells. It showed that not only protein but also peptide could be efficiently entrapped in RBCs by a controlled lysis/resealing process. In both antigen models, the administration of a small quantity of antigen-loaded in RBCs combined with poly(I:C) induced an antigen-specific T-cell response and the control of tumor growth in mice, whereas the injection of the same quantity of free antigen did not. The intensity of the T-cell response was dependent on the concentrations of antigen entrapped and the treatment performed on the RBC membrane to improve antigen delivery. In summary, these results support the use of RBCs as an antigen delivery system for a powerful cancer immunotherapy approach.

Magali Cremel and colleagues (2013) investigated the effects of ovalbumin (OVA)-loaded RBCs on the humoral response in mice. They found that OVA-loaded RBCs significantly reduced the humoral response compared to OVA alone. The state of tolerance induced by RBCs was long-lasting, antigen-specific, and sufficiently robust to withstand immunization with antigen mixed with cholera toxin adjuvant 133.

Thymidine phosphorylase (TP) is an enzyme that is overexpressed in many types of cancer cells. It catalyzes the conversion of thymidine to deoxyuridine monophosphate (dUMP), which is then used to synthesize DNA. By targeting TP, it is possible to inhibit DNA synthesis and kill cancer cells. There are several ways to target TP 134. One approach is to use TP inhibitors, which are molecules that bind to TP and prevent it from catalyzing the conversion of thymidine to dUMP. TP inhibitors are currently being developed as cancer drugs. Another approach is to use TP-deficient cells as cancer vaccines. These cells are engineered to lack TP, which makes them unable to survive in the body. When they are injected into a patient, they are recognized by the immune system as foreign cells and are killed. This triggers the production of antibodies against TP, which can then kill cancer cells that express TP.

The study of erythrocyte-encapsulated thymidine phosphorylase (EE-TP) in treating mitochondrial neuro gastrointestinal encephalomyopathy (MNGIE) involved three adult patients with MNGIE who received intravenous EE-TP at increasing doses over a period of 4 weeks. The results showed that EE-TP was well tolerated and resulted in significant reductions in the levels of thymidine and deoxyuridine, two of the harmful metabolites associated with MNGIE 135. Preclinical studies were conducted to assess the safety of EE-TP. The studies involved mice and dogs that were administered EE-TP by intravenous bolus injection 136. The results showed that EE-TP was well tolerated in both species. However, some infusion-related reactions were observed in dogs, including transient clinical signs, such as lethargy and decreased activity. Orphan Technologies utilizes thymidine phosphorylase to significantly improve the cure of rare homocysteine and related diseases.

WTX-212, this product is a novel erythrocyte-anti-PD-1 antibody conjugate that is being developed by Westlake Therapeutics. WTX-212 is being developed based on the REDx technology platform. REDx is a technology that allows for the precise control of the distribution of T cell activation ligands on the membrane of RBCs. With antitumor efficacy in models of acquired resistance to immune checkpoint inhibitors. The therapy of WTX-212 comprises autologous engineered RBCs administered as an infusion. WTX-212 is being developed based on the RBC drug technology platform REDx and acts by expressing serine protein kinase B-Raf-g469v mutated protein and it is now in clinical trial Phase 1.

Dex 21-P, this product uses RBC low permeability load technology to load dexamethasone sodium phosphate into patient RBCs, to cure ataxia telangiectasia and is in phase III clinical trials by EryDel (Italy) 137. This platform extends the circulation time of drugs to 86 days.

Eryaspase, the comerial name of GRASPA (L-asparaginase) 138, this product uses enzymes to attach drugs or genes to the surface of RBCs from ERYCAPS platform, which is another Erytech company. This allows the drugs or genes to be delivered to cancer cells more effectively. The drug eryaspase is designed to treat acute myeloid leukemia, acute lymphocytic leukemia, pancreatic cancer, and non-Hodgkin's lymphoma. ERYCAPS technology can package a wide range of molecules from 1 kD to 500 kD.

CD34^+^ hematopoietic stem cells, Rubius used bioengineering technology on CD34^+^ hematopoietic stem cells from healthy O-type donors for cell therapy products, such as RTX-134 for PKU (phenylketonuria), RTX-240 for AML, and RTX-321 (RTX-aAPC) for HPV 16^+^ cancer. Rubius Therapeutics has developed in-stock RTX-240 through I/II phase clinical trials for r/r AML (refractory and recurrent AML) patients and for solid tumors. RTX-240 coexpresses 4-1BBL (4-1 BB ligand) and IL-15TP on RBCs, and these two ligands can induce the immune system of patients. 4-1BBL drives NK cell and T cell proliferation and activation and produces IFNγ, while as the fusion of IL-15 and IL-15 receptor α, IL-15TP can bridge innate immunity and acquired immunity. However, Rubius Therapeutics cut off the remaining research on RTX-240 and RTX-224 due to insufficient curacy.

In addition, Anokion technology takes advantage of the specific protein glycophorin A on the surface of RBCs, and is links with self-immune disease-related antigens such as OVA, these antigen-combined RBCs modulate immune tolerance to avoid unnecessary immune responses 139. This allows the antigens to be delivered to cancer cells more effectively. Cello Therapeutics develops biomimetic cell membrane-coated nanoparticles that can be used to deliver cancer drugs, including chemotherapy agents, immunotherapy agents, or mRNA. This technology uses the membrane of RBCs to enwrap nanoparticles and increase the drug payload and possibilities of targeted delivery. This allows the nanoparticles to be delivered to cancer cells more effectively. Nanoparticle-based drug carriers have obtained great therapeutic potential 140.

### CD47-inspired cancer immunotherapy

CD47 is a cell-surface molecule that is highly expressed in RBCs and in cancer cells 141, 142. It can send a message of "do not eat me" to hide immune system surveillance and promote immune evasion by engaging signal-regulatory protein alpha (SIRPα), because CD47-SIRPa originally serves as an inhibitory receptor on macrophages 143. Kong F. *et al.* (2016) 141 discussed the promising therapeutic application of anti-CD47 antibodies for eliminating tumor cells. Vonderheide R H. *et al.* (2015) reviewed the blockage of CD47 as an immunotherapy target not only by phagocytosis of macrophages but also by T cells and dendritic cells 144. A report on the immune response by McCracken M N *et al.* (2015) showed that monotherapy focused on CD47: SIRP-α engagement can modulate antitumor T cells *in vivo* 145. Weiskopf K *et al.* (2016) used the CD47 immunotherapy method to treat small lung cancer patients 146.

### Sialic acid-inspired cancer immunotherapy

Carbohydrate vaccines for cancer immunotherapy are based on self-antigens that are expressed on cancer cells and can be recognized by the immune system. These vaccines can induce an immune response that can kill cancer cells 147. Sialyl Tn (sTn) antigen is a sialylated disaccharide that is expressed on some cancer cells. It can be conjugated to keyhole limpet hemocyanin (KLH), which is a carrier protein that helps the immune system to recognize the antigen. Two types of sTn-KLH conjugates have been developed: N-iso-butanoyl sTn-KLH and N-phenylacetyl sTn-KLH. Both conjugates induce an immune response dependent on T cells. However, N-phenylacetyl sTn-KLH is more suitable for developing cancer immunotherapy, especially vaccines 148. sTn is expressed on three of 10 types of normal epithelia. However, it is overexpressed in some cancer cells, such as prostate cancer cells. This makes it a good target for cancer immunotherapy. Zhang et al. (1998) studied the methods that could be used to select antigens for cancer immunotherapy. They found that sTn was a good target for prostate cancer because it was overexpressed on these cells and it induced an immune response that could kill cancer cells 149. And Jicheng Wu *et al.* developed tumor-targeting molecules that can selectively remove sialoglycans from cancer cells. They found that this process, called desialylation, triggers CAR-iMac cells to become activated and more effective at killing cancer cells. This suggests that cancer cell desialylation is a promising new approach to improving the effectiveness of solid tumor cellular immunotherapy 150.

### Erythrocyte-driven immune targeting (EDIT)

Erythrocyte-driven immune targeting (EDIT) is a strategy that presents nanoparticles from the surface of erythrocytes to the antigen-presenting cells (APCs) in the spleen. Researchers take advantage of the natural antigen-presenting function of RBCs in capturing and presenting pathogens in the blood. This innate immune function of RBCs can be leveraged to enhance the immune response against antigenic nanoparticles adsorbed on the erythrocyte surface. For example, EDIT has been shown to improve antibody response against the antigen, increase central memory T cell response, and decrease regulatory T cell response, which can slow down tumor progression 104. However, senescent or apoptotic erythrocytes can also be used to achieve antigen-specific immune tolerance. This is because these cells are not as effective at presenting antigens to APCs as healthy erythrocytes. This makes it crucial to control the type of erythrocytes used in EDIT for tumor immunotherapy 99.

## Clinical progress of drug-loaded RBCs

RBCs have been the subject of extensive research as drug delivery systems for the past 5 decades due to their potential to modulate the pharmacokinetic, pharmacodynamic, and biological properties of the drugs they deliver. In recent years, a small number of RBC-based drug delivery systems (DDSs) have been introduced to clinical trials 77. The review from Siyu Wang et al. summarized RBC-NPs used for delivering chemotherapy therapeutics for cancer treatment 151. The clinical trials summarized in Table 2 demonstrate the use of RBCs as drug delivery systems (DDSs) for both interventional and diagnostic applications.

We can see from the table that more efforts are made in the clinic to cure diseases, while only a few attempts are made for diagnosis. The success rate for new applications is very rare. However, the ultimate goal of discovering and developing drug delivery systems (DDSs) is to bring them to the clinical stage and achieve large-scale industrial production. Most studies on drug-loaded RBCs are still in the preclinical stage, but some formulations have undergone clinical research and may be used to treat patients in the near future. The development of DDSs is a long and complex process, but it has the potential to revolutionize the way we treat diseases.

RBCs are a promising platform for DDSs, but there are still many challenges that need to be addressed before they can be used in the clinic. Despite the challenges, the development of DDSs is an exciting field with the potential to improve the lives of millions of people.

## The factors affecting the aging and clearance of RBCs

This process of RBC aging and clearance is essential for maintaining the health of the blood and preventing the accumulation of damaged RBCs.There are a number of factors that contribute to cell aging. As a kind of cell in the bloodstream, RBCs are constantly exposed to oxygen, which can lead to the production of free radicals. Free radicals are unstable molecules that can damage RBCs and other cells in the body. Another factor that contributes to RBC aging is glycation. Glucose can bind to proteins and lipids in RBCs, forming compounds called advanced glycation end products (AGEs). AGEs can damage RBCs and make them more susceptible to clearance by the spleen and liver.

Circulating around the blood vessels and carrying oxygen are prone to turn out oxidation for RBCs, as the reduced forms of nicotinamide-adenine dinucleotide (NAD) and glutathione (GSH) are involved in their energy sources, and excess O^-^ affects the structure of the membrane and causes physical damage to the spleen, liver, and narrow capillaries. The membrane change of normal RBCs can lead to recognition failure affinity by autologous immunoglobulin G (IgG) and complements involved in the phagocytosis of RBCs, as well as abnormal hemoglobin, Fe^2+^, band 3, and phosphatidylserine (PS) levels that influence RBC structure and functions. Lysed membrane, released hemoglobin, and heme, a coordination complex consisting of an iron ion coordinated to a porphyrin acting as a tetradentate ligand, and to one or two axial ligands, can be cleared up by an immune complex, but effete RBCs (senescent RBCs and oxidatively damaged RBCs) have different phagocytosis mechanisms. Now, we summarize the related factors that lead to RBC aging and phagocytosis.

### Formation and exposure of clearance-related molecules

Band 3 (anion channel) is the major erythrocyte membrane-spanning protein and the integrating membrane protein. It is also known as AE1 (anion exchanger) and is anchored to the membrane skeleton as a tetramer. The mobile fraction of band 3 forms dimers and transports bicarbonate (HCO_3_^-^) from tissues around the body to the lungs. Several articles have reviewed naturally occurring antibodies (NAbs) (also called autologous IgG) that have an affinity for band 3 152, 153. When conditions become harsh, such as with oxidative damage, band 3 dimers can form oligomers. NAbs can easily bind to band 3 oligomers and then induce the immune system to phagocytose the cells. Band 3 protein clustering on human erythrocytes also promotes the binding of NAbs and anti-spectrin antibodies 154.

The complement system also has important effects on the clearance of RBCs. H.U. Lutz et al. (2004) reviewed detailed information on this topic in 2004 155. In addition to oxidative stress, band 3 clustering can also be caused by hemoglobin and exoplasmic crosslinking. Hemochrome is formed when hemoglobin is denatured. Hemochrome can then crosslink band 3 proteins into clusters. These clusters become the recognition sites for cell-senescent antibodies. Diffusion-mediated oligomerization can also lead to exoplasmic crosslinking, band 3 clustering, and bivalent binding of anti-band 3 naturally occurring antibodies (NAbs). Complement proteins can then deposit on these clusters.

**Figure 9:**
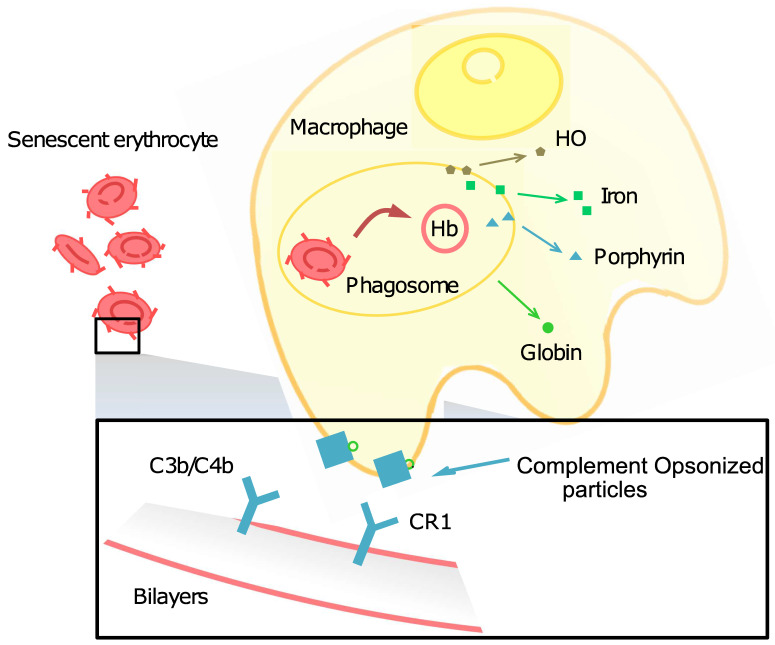
Erythrocytes help phagocytose complements, erythrocytes, and complement-opsonized particles interact through C3b/C4b (CR1) and are phagocytosed by macrophages, and erythrocytes are decomposed by phagosomes to release iron, porphyrin, globin, and HO (heme oxygenase) for recycling.

RBCs circulating around the body not only participate in gas exchange but also play an immune adherence role for later phagocytosis by interacting with complement receptor 1 (CR1, CD35), also known as complement 3b/4b (C3b/C4b). RBCs use CR1 to bind circulating complement-opsonized particles 156. RBCs use CR1 to bind circulating complement-opsonized particles, such as microbes, immune complexes, and self-generated inflammatory particle adherent complexes. They then remove and transfer these particles to resident macrophages in the liver and spleen for clearance 17 (Figure 9).

Phosphatidylserine (PS) is an important phospholipid membrane component. Normal phospholipids can move freely within the membrane, but PS is restricted to the inner leaflet by flippases. Exposure of PS on the outer leaflet of RBCs, accompanied by elevated intracellular calcium (Ca^2+^), results in opsonization and engulfment by the mononuclear phagocyte system, leading to RBC clearance 157.

### Loss or conformational change of self-marker molecules

#### CD47 as a self-marker molecule

CD47 is an integrin-associated protein that is associated with human Rhesus antigens. It has a molecular weight of 47-52 kDa. The cytosolic soluble segment of CD47 on the surface of the membrane can interact with the inhibitory receptor signal regulatory protein alpha (SIRPα) on macrophages to confirm self-identity. This interaction prevents phagocytosis, so CD47 is called a self-marker protein on mammalian RBCs. When CD47 is oxidized, it can crosslink into dimers. These dimers can interact with SIRPα on macrophages, which sends a signal that triggers phagocytosis. This is why CD47^-/-^ RBCs (RBCs that lack CD47) are cleared more rapidly than wild-type RBCs. Oldenborg *et al.* (2000, 2001) studied CD47^-/-^ RBCs in wild and CD47^-/-^ mice. They found that CD47^-/-^ RBCs were cleared very rapidly in wild mice, and that CD47-/- aggravated anemia in CD47^-/-^C57BL/6 mice 143. H.U. Lutz (2004) concluded that increased opsonization and a lower amount of CD47 enhanced phagocytosis. This means that if RBCs are coated with antibodies or other proteins (opsonization), or if they have fewer CD47 molecules, they are more likely to be phagocytosed 155.

CD47 is generally lost as RBCs circulate around the body. The inhibition of phagocytosis by CD47 molecules may have a dose-dependent effect, meaning that the amount of CD47 on the RBCs affects the effectiveness of its inhibitory signal 158. For example, senescent RBCs show a very low content of CD47 on the membrane. CD47 is also affected by RBC aging and oxidation during circulation 159. Additionally, the inhibition efficiency depends on the binding strength of the CD47-SIRPα interaction.

The "do not eat me" signal sent by CD47 can inhibit the actomyosin cytoskeleton of macrophages, which is a network of proteins that helps the macrophage to engulf its target. Nisha G. Sosale et al. (2015) identified that phagocytosis may be driven by the typical rigidity of microbes and many synthetic materials. This insight has already been applied to basic research on how macrophages engulf cancer cells or enhance the circulation time of nanoparticles in the body 160. The adhesive process of macrophage engulfment is mediated by myosin-II, a protein that helps to contract the cytoskeleton. Activation of myosin II makes the macrophage phagocytic synapses rigid. If CD47 sends the "do not eat me" signal, myosin will be inhibited and the macrophage will not be able to engulf its target. However, the rigidity of phagocytosed RBCs can hyperactivate myosin, overwhelming the "self" signal of CD47 to macrophages and causing them to engulf the RBCs.

#### Sialic acid gets influenced by RBCs aging

Sialic acids on the RBC membrane are mostly conjugated to galactose (Gal) residues. These sialic acids act as modifiers of proteins, lipids, and glycoproteins. If sialic acids are stripped off, the Gal residues are exposed to various molecules. This can lead to the sequestration of RBCs from the circulation system by macrophages 161.

Siglecs are lectins that belong to the IgG superfamily and bind to N-acetylneuraminic acid residues that link alpha 3 or alpha 6 to galactose. Human RBCs bind to immunoreceptor tyrosine-based inhibitory motif (ITIM)-carrying siglecs in macrophages and prevent the engulfment of sialic acid-carrying cells, which makes sialic acid a self-marker molecule 155.

Aminoff *et al.* (1988) defined senescent RBCs as "RBCs do show the greatest number of time-dependent changes" 162, and D. Bratosin *et al.* (1998) depicted it more vividly: "senescent RBCs represent the erythrocyte population which has been condemned to death and which thus has one foot in the grave". When treated with neuraminidase, phagocytosed and adherent RBCs by monocytes sustained a higher percentage than young RBCs and more membrane-bound IgG 163. Mohammad and his colleagues (2012) also found that the sialic acid content in the erythrocyte membrane decreases significantly during RBC aging in human blood, while hydroperoxides can lead to a significant increase in lipid peroxidation 21. In most cases, desialylation of sialoglycoprotein directly leads to exposure to galactoside, which can be recognized by galactoside receptors in the liver. Loss of glycocalyx (i.e., loss of negative charges) leads to the increased sodium sensitivity of erythrocytes 20, and lack of sialic acid in RBCs is prone to affinity to complement and autologous IgG, and then activates the phagocytic process. However, Biondi *et al.* (2003) found that desialylation was not involved in the increased removal of erythrocytes observed in elderly individuals 164.

#### Other membrane proteins related to RBC clearance

Another cluster of differentiation (CD) molecules related to RBC clearance is CD44 on the erythrocyte membrane. CD44 is a phagocytic receptor 165 and a surface marker in human erythrocytes. It is involved in the inflammatory response, helping to recruit immune cells to the site of inflammation. Oxidative modification of membrane-cytoskeleton proteins can change the levels of CD44 and its characteristics under the changed physicochemical parameters of the cellular environment. This may be due to changes in the protein-protein interactions in the cell membrane. In drug-loaded RBCs, CD44 plays a key role in maintaining the stability of the cell membrane 166. Damage to the cell membrane can affect the ability of RBCs to bind to other cells and tissues, leading to problems such as anemia, rheumatoid arthritis, and multiple sclerosis. CD44 of desialylated or aged erythrocytes interacting with hyaluronic acid on vascular endothelia can facilitate the sequestration of RBCs., as in drug-loaded RBCs, CD44 plays a key role in the stability of cell membrane 167.

Glycophorin A (GPA) is the major transmembrane sialoglycoprotein of RBCs 168 and specific to erythrocyte membranes 169. It has been shown to contribute to the expression of the MN and Wright blood group antigens, to act as a receptor for the malaria parasite Plasmodium falciparum 170 and Sendai virus, and along with the anion transporter, band 3, may contribute to the mechanical properties of the RBC membrane. GPA protein is involved in the transport of oxygen and carbon dioxide in the blood. It is also involved in the immune response, helping to protect the body from infection by identifying and targeting foreign cells. GPA protein is also involved in the clearance of damaged or infected RBCs from the blood 171. Without GPA protein, the immune system would not be able to function properly. The GPA-targeted ERY1 ligand enhances binding without impacting cellular function 172.

### Change of membrane properties with blood rheology

Blood rheology is also considered to be related to RBC aging. The rheology of blood is influenced by two important factors in erythrocytes: aggregability and deformability. These two factors reflect the key determinants of blood viscosity at high and low shear rates. Simmonds *et al.* (2013) found that blood rheology and aging cooperated together 173. Fens and Storm* et al.* (2010) measured the phagocytosis of opsonized erythrocytes with reduced deformability by angiogenic growth factor-activated endothelial cells and found a significant increase in whole-blood viscosity (WBV) 174. Harisa *et al.* (2015) acclaimed an oxidative stress-mediated mechanism, and oxidative stress led to less WBV 23. The reduced deformability of RBCs over time was associated with a poor outcome in septic patients. This was due to changes in the inner constitutions of band 3, outer exposed phosphatidylserine (PS), and lactadherin-bridging molecules. Li *et al.* (2010) studied the factors associated with increasing blood viscosity 175. Malondialdehyde (MDA) is a lipid oxidation product that is used as a biochemical indicator to measure the degree of lipid oxidation. MDA is formed when free radicals attack polyunsaturated fatty acids in cell membranes. This can lead to the oxidation of membrane proteins and glycoproteins, with a high level of MDA and a low level of GSH indicating increased oxidative stress, which can damage cells and tissues 176.

Less deformability may occur in effete RBCs, and it may affect the deformability of surrounding RBCs' 177. Effects RBCs are old, worn-out RBCs that have lost their ability to deform. They are removed from the circulation through a process called mechanical trapping in the spleen. Deformability is also positively affected by complement receptor 1 (CR1). A CR1-dependent increase in membrane deformability could facilitate the transfer of CR1-bound particles from RBCs to hepatic and splenic phagocytes 156. Angiogenic endothelial cells enhance erythrophagocytosis by loss of erythrocyte deformability 174.

The clearance of RBCs is affected by their shape. Experiments have shown that rigid but more rounded RBC stomatocytes signaled "self" more than rigid RBC discocytes, highlighting the effect of shape. The physical properties of phagocytic targets can thus modulate "self" signaling, as they seem to be relevant to the splenic clearance of rigid RBCs after storage or the clearance of rigid pathological cells such as sickle and thalassemic RBCs 160. While CD47^-/-^ cells are not inherently responsible for abnormalities in cell shape 21, sphericity and surface loss can increase splenic entrapment in *Plasmodium falciparum*-infected RBCs 178.

### RBCs phagocytosis mainly in liver and spleen

Sequestration of senescent RBCs is thought to take place primarily in reticuloendothelial cells (RE cells) in the spleen, liver, and bone marrow. RE cells are cells that phagocytose (engulf and destroy) foreign particles and cells.

The liver is a large immune organ whose sinusoidal cells are often referred to as Kupffer cells. Kupffer cells in the liver are major residential hepatic immune cells; approximately 25% of nonhepatocyte cells are Kupffer cells, second to endothelial cells (approximately 50% of nonhepatocyte cells in the whole liver) 179, and Kupffer cells are a distinct population of RBC-phagocytizing cells in the liver 180. Blood in the liver will go through the portal vein. As galectins in the liver have an affinity for desialylated gal residues, gal-exposing RBCs are phagocytosed by Kupffer cells. Therefore, the liver is the primary organ to remove damaged RBCs.

The spleen serves as the largest filter of blood in the human body. The structure of the spleen not only adapts to remove older erythrocytes from the circulation but also efficiently removes blood-borne microorganisms and cellular debris 181. The structure and function of the spleen make special contributions to blood circulation. Blood in the spleen needs to undergo two kinds of circulation: one is that RBCs traverse venous sinuses bypassing the red pulp, so-called "closed circulation". During this process, the meshwork in the red pulp ascertains the structural and mechanical quality of RBCs, retains young and normal RBCs, and removes old and abnormal RBCs; the other is called “open circulation”, during which the remaining blood goes through the reticular meshwork, then the microcirculation 182. Safeukei and colleagues (1996, 2008) designed a method for perfusing recently removed human spleens with tagged RBCs called the "eosin-maleimide test" 183. This test is expected to determine the key determinate that makes the spleen clear the effete RBCs, and the "eosin-5-maleimide test" (eosin-5-maleimide binding is a diagnostic test for hereditary spherocytosis) has been applied in clinical practice 184.

### The clearance of drug delivery RBCs

The properties of RBCs are easily altered by drug loading 185, 186, which can affect the clearance process and lead to unprecedented changes in the pharmacokinetics, pharmacodynamics, and immunogenicity of the drugs. The clearance of drug delivery RBCs will affect the circulation time and target site of the drugs, which have a significant impact on the way that the drugs are metabolized, distributed, and eliminated by the body. Accordingly, some specific, salient parameters controlling behavior of RBC/DDS are defined in the body 83.

Despite transmitting infections or choosing the wrong blood type, for it is a complex and sterile process will lead to clearance, there are many factors that contribute to the clearance of drug delivery RBCs. These factors include the type of drug that is being delivered, the method used to load the drug onto the RBCs, the properties of the RBC membrane, the immune response to the drug-loaded RBCs.

The type of drug that is being delivered can affect the clearance of drug delivery RBCs in a number of ways. For example, drugs that are more easily metabolized by the body tend to be cleared more quickly than drugs that are not easily metabolized. This is because the body can break down and remove these drugs more easily.

The method used to load the drug onto the RBCs can also affect the clearance of drug delivery RBCs. In order to make sure the high quality of RBCs, any crude method for preparation should be avoid, otherwise, these drug-delivery RBCs will rapidly degrade in the bloodstream. For example, drug-delivery RBCs that are loaded using a physical method, such as encapsulation, tend to be cleared more slowly than drug-delivery RBCs that are loaded using a chemical method, such as conjugation. This is because physical methods of loading drugs onto RBCs do not damage the RBC membrane as much as chemical methods 187. Damage to the RBC membrane can make the RBCs more susceptible to clearance by the immune system.

The properties of the RBC membrane can also affect the clearance of drug-delivery RBCs. Normally, RBCs with a more negatively charged membrane tend to be cleared more slowly than RBCs with a less negatively charged membrane. This is because the negatively charged membrane repels the immune cells that would otherwise clear the RBCs. The function of sialic acids is very important in this regard. Sialic acids are negatively charged molecules that are found on the surface of RBCs. They help to protect RBCs from being cleared by the immune system.

The immune response to the drug-loaded RBCs can also affect their clearance. The drug-loaded on RBCs that are rejected by the immune system will be cleared more quickly.

Understanding the factors that affect clearance can help researchers develop methods to prolong the circling time of drug delivery RBCs. Researchers are also working on developing drugs that can suppress the immune response to drug-delivery RBCs. This would help to prevent the drug delivery RBCs from being cleared by the immune system too quickly and make them more effective for delivering drugs to patients.

In addition, we have analyzed key molecules on the membrane, such as CD47 and sialic acids. These molecules are important for maintaining the compatibility of RBCs. CD47 is a "don't eat me" molecule that helps to prevent immune cells from attacking RBCs. Sialic acids are negatively charged molecules that help to repel immune cells (see Section “The membrane”). There will be more choices to mimic when the biology and structure characteristics of RBCs are fully understood.

## Conclusion and perspectives

RBC engineering provides numerous ways to optimize clinical curation and diagnosis. With cancer immunotherapy being widely used, RBCs as a vector will be increasingly popular due to their compatibility and long circulation time. RBCs could help to solve many difficulties in the biotechnology and biomedical fields in the future. We reviewed different methods that have been used to load drugs onto RBCs, and the applications of RBC-based DDS. Furthermore, the challenges and future directions of RBC-based DDS are key points to explore.

The research on U-RBCs is a promising field with the potential to revolutionize blood transfusion medicine. U-RBCs could also have other benefits. For example, they could be used to treat anemia, sickle cell disease, and other blood disorders. They could also be used to deliver drugs and other therapies to the body. However, with the discovery of new blood group antigen profiles from different geographic regions 188, clinics need to further develop rational preparation of whole blood or blood components and efficient bioprocesses 189. With continued research, it is possible that these products could be available for clinical use in the near future.

The application of RBCs in drug delivery system takes advantage of the quality that RBCs are abundant, easily accessible, and have a long circulation time in the blood. In addition, RBCs are able to target specific tissues and cells, making them ideal for the delivery of drugs to hard-to-reach areas. Despite DDS of RBCs improved the loading efficiency and traveling time of drug, as well as the dose and efficacy of drug release, DDS of RBCs still have challenges in maintaining the function and properties of RBCs, avoiding aggregation and blood clotting, decreasing fatigue, senescence, and damage. Many researchers investigated on loading and relaxation dynamics of an RBC, when mechanical load on the membrane, the deformation and rotational contributions were induced and Guglietta F *et al.* figured out the imposition/cessation of external mechanical loads on RBCs 190. The viscoelastic response of RBCs during loading is involved in traits like reduced deformability, increased membrane viscosity, and change in cell shape, causing substantial changes in the overall hemodynamics 191.

Drug-loaded RBCs are most commonly administered via vascular injection, but they have also been studied for oral administration 192. Studies on oral administration of drug-loaded RBCs are still in their early stages. Oral administration of drug-loaded RBCs could potentially change the immune response to xenogeneic cells 193, 194. Oral administration of drug-loaded RBCs could benefit patients who cannot tolerate injections or need to take multiple medications. However, there are challenges such as protecting RBCs from the harsh environment of the stomach like varying pH (highly acidic stomach) and the presence of enzymes, and controlling the release of drugs to avoid toxicity, as well as the first-pass effect in the liver and the intestinal barrier to drug absorption 195. In addition, another novel application of RBCs for therapy is RBC-based hydrogel. RBC hydrogel is a gel derived from RBCs and utilized in applications such as tissue regeneration and cancer photo-immunotherapy. This hydrogel capitalizes on the platelet activation and photothermal properties of RBCs. Ziying Fei et al. developed an injectable RBC-based gel for cancer therapy. Upon exposure to near-infrared (NIR) laser light, this hydrogel effectively releases tumor-associated antigens (TAAs), thereby triggering a sustained immune response that leads to the eradication of tumor 196.

We list above many factors that contribute to effete RBCs, and the determinates of senescent RBCs, while the long circulation time *in vivo* benefits from the structural completeness of the cell membrane and high deformability, as well as biconcave shape; and the marker of self-molecules such as CD47 and sialic acids get its passport all along the way. Some antioxidant complexes also sustain membrane completeness. It is known to us all that effete RBCs have multiple regressive characteristics, which will aggressively affect the traveling time of RBCs. While senescent cells are phagocytosed and cleared by the liver, spleen, and other reticuloendothelial systems. The targeting of senescent cells in the liver and spleen is a potential therapeutic strategy for a variety of diseases. Researchers are currently investigating ways to target senescent cells to certain organs such as the liver and spleen 104.

Mathematical models show surface area and membrane shear modulus are important parameters to evaluate the aging status 197. Travis Nemkov et al. (2020) found an alternative way for some RBCs to overcome stress conditions, by decoding the metabolic landscape 198. The use of these models, techniques, and research on the membrane property index will make it more convenient to evaluate the state of RBCs and drug-loaded RBCs. It will also be easier to find optimal conditions for sustaining the suitable condition for RBCs as a vector for drug delivery. With these models, techniques, and research on the content of the membrane, the evaluation on the state of RBCs and drug-loaded RBCs will be convenient, and easy to find optimal conditions for sustaining the suitable condition for RBCs as a vector for drug delivery. Future studies could use Uncertainty quantification (UQ) techniques and computational model updating to improve the predictive capacity of models and assist in more reliable treatment planning and outcome assessment 199.

## Figures and Tables

**Figure 4:**
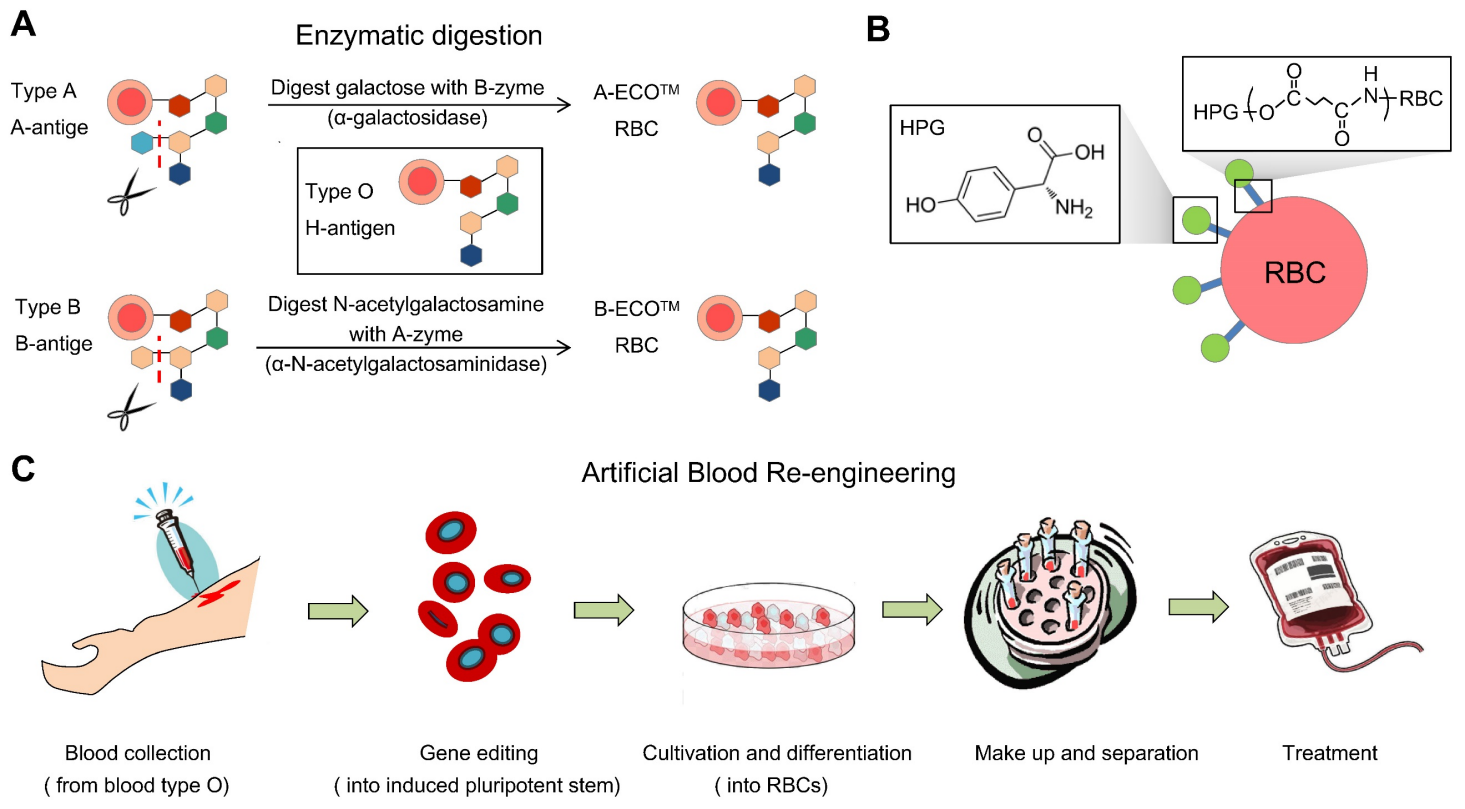
Common means to obtain universal RBCs for transfusion. (A) Preparation of enzyme-converted group O (ECO) blood cells by enzyme digestion. (B) Shielding antigens of the membrane surface from donors. (C). Inducing RBCs from iPS cells, gene editing, and adjusting the culture procedure to control the blood types.

**Table 1:** The specific chain structure of human RBC antigens, antibodies, and their responsive enzymes for enzyme-converted group O (ECO) RBCs.

Phenotypes	Structure of dominant immune epitope	Antibodies in sera	Glycosyltransferases
A type	α-3-N-acetylgalactosamine	anti-B	α-3-N-acetylgalactosaminyl
Subgroup A1	A and repetitive A	anti-B	
Subgroup A2	A	anti-B and anti-A1	
B type	α-3-galactose	anti-A and anti-A1	α-3-galactosyl
AB type	A and B	No anti-A or anti-B	
A1B	A, repetitive A and B	No anti-A or anti-B	
A2B	A and B	No anti-A or anti-B anti-A1	
O type	No	anti-A and anti-B	

(Revised from Schachter et al. (1973) 29 and Clausen, Henrik, et al. (1985) 30)*.*

**Table 2:** List of clinical trials for RBC DDS (retrieved from www.clinicaltrials.gov).

Study Type	Interventional						
Product	Carried drugs	Loading method	Conditions	Study Status	Sponsor	Phases	ClinicalTrials.gov Identifier
Eryaspase	GRASPA(L-asparaginase)	Encapsulated-Hypotonic dialysis	Acute Lymphoblastic Leukemia	Completed	ERYtech Pharma	Phase 2	NCT01523782
Eryaspase	GRASPA(L-asparaginase)	Encapsulated-Hypotonic dialysis	Acute Lymphoblastic Leukemia, in Relapse	Completed	ERYtech Pharma	Phase 2/3	NCT01518517
Eryaspase	GRASPA(L-asparaginase)	Encapsulated-Hypotonic dialysis	Acute Myeloid Leukemia	Completed	ERYtech Pharma	Phase 2	NCT01810705
Eryaspase	GRASPA(L-asparaginase)	Encapsulated-Hypotonic dialysis	Triple Negative Breast Cancer	Terminated	ERYtech Pharma	Phase 2/3	NCT03674242
Eryaspase	GRASPA(L-asparaginase)	Encapsulated-Hypotonic dialysis	Pancreatic Adenocarcinoma	Completed	ERYtech Pharma	Phase 3	NCT03665441
Eryaspase	GRASPA(L-asparaginase)	Encapsulated-Hypotonic dialysis	Locally advanced pancreatic ductal adenocarcinoma, metastatic pancreatic ductal adenocarcinoma	Active, not recruiting	ERYtech Pharma	Phase 1	NCT04292743
Dex 21-p	Dexamethasone 21-Phosphate	Osmotic loading	Atherosclerosis, Acute-Phase Reaction	Unknown	University of Rome Tor Vergata	Phase 4	NCT00484965
Dex 21-p	Dexamethasone 21-Phosphate	Osmotic loading	Ulcerative Colitis	Unknown	Casa Sollievo della Sofferenza IRCCS	Phase 2	NCT01171807
EryDex	Dexamethasone 21-Phosphate	Osmotic loading	Crohn's Disease	Terminated	EryDel	Phase 3	NCT01277289
EryDex	Dexamethasone 21-Phosphate	Osmotic loading	Healthy	Completed	EryDel	Phase 1	NCT01925859
EryDex	Dexamethasone 21-Phosphate	Osmotic loading	Ataxia telangiectasia	Recruiting	EryDel	Phase 3	NCT03563053
IEDAT01	Dexamethasone 21-Phosphate	Osmotic loading	Nervous System Disorder, Genetic Syndrome	Completed	Erydel	Phase 2	NCT01255358
WTX212	engineered RBCs with PD-1 inhibitor pembrolizumab	Genetic engineering	Cancer, Solid Tumor, Hematologic Malignancy	Recruiting	Westlake Therapeutics	Phase 1	NCT05707325
Thymidine phosphorylase	Thymidine phosphorylase	Encapsulated-Freely diffuse	Mitochondrial neurogastrointestinl encephalomyopathy	Active, not recruiting	St George's, University of London	Phase 2	NCT03866954
RTX-134	allogeneic human RBCs expressing the AvPAL	Genetic engineering	Phenylketonuria	Terminated on 11/30/22	Rubius therapeutics	Phase 1	NCT04110496
RTX-240	Engineered RBCs co-expressing 4-1BBL and IL-15TP	Genetic engineering	Solid Tumor, AML Adult	Terminated on 11/30/22	Rubius Therapeutics	Phase 1/2	NCT04372706
RTX-321	Engineered RBCs co-expressing 4-1BBL, IL-12, and HPV-16 Antigen	Genetic engineering	Cervical Cancer, Head and Neck Cancer, Anal Cancer	Terminated on 11/30/22	Rubius Therapeutics	Phase 1	NCT04672980
RTX-224	Engineered RBCs co-expressing both 4-1BBL and IL-12	Genetic engineering	Non-Small Cell Lung Cancer, Cutaneous Melanoma, Head and Neck Squamous Cell Carcinoma, Urothelial Carcinoma, Triple-Negative Breast Cancer	Terminated on 11/30/22	Rubius Therapeutics	Phase 1/2	NCT05219578
SQZ-AAC-HPV	Immunogenic epitopes of human papillomavirus strain 16+	Microfluidic-based cell squeeze	Metastatic HPV16 + solid tumors	Recruiting	SQZ Biotechnologies	Phase 1	NCT04892043
Ery-apoB	Erythrocyte-bound apolipoprotein B	Transgenic expression	Hyperlipidemia, Atherosclerosis	Completed	Sint Franciscus Gasthuis	Not Applicable	NCT01634906
MTX	Autologous Erythrocytes Derived Microparticles Packaging Methotrexate	Encapsulated-Hypotonic dialysis	Malignant Ascites	Unknown	Hui ting Xu, MD	Phase 1/2	NCT03230708
Study Type	Diagnostic						
indocyanine green (ICG)-loaded erythrocytes	ICG	Encapsulated-Hypotonic dialysis	Retinal Disease	Withdrawn	Northwell Health	Not Applicable	NCT02445001
Biotin-Labeled RBCs	Biotin	Bio-Labeled	Healthy Volunteers	Terminated	Gladwin, Mark, MD	Phase 2	NCT03364686
Biotin-Labeled RBCs	Biotin	Bio-Labeled	Anemia	Completed	Emory University	Phase 1	NCT02757898
FDG-labeled Human Erythrocytes	FDG	Bio-Labeled	Breast Cancer|Breast Cancer, Female|Breast Cancer, Male|Breast Neoplasms	Withdrawn	H. Lee Moffitt Cancer Center and Research Institute	Early Phase 1	NCT03295695

Note: The specific information can be searched in https://clinicaltrials.gov by ClinicalTrials.gov Identifier
